# Charge-density reduction promotes ribozyme activity in RNA–peptide coacervates via RNA fluidization and magnesium partitioning

**DOI:** 10.1038/s41557-022-00890-8

**Published:** 2022-02-14

**Authors:** Juan M. Iglesias-Artola, Björn Drobot, Mrityunjoy Kar, Anatol W. Fritsch, Hannes Mutschler, T.-Y. Dora Tang, Moritz Kreysing

**Affiliations:** 1 Max Planck Institute of Molecular Cell Biology and Genetics, Dresden, Germany; 2 Helmholtz Zentrum Dresden Rossendorf, Dresden, Germany; 3 Center for Systems Biology Dresden, Dresden, Germany; 4 Department of Chemistry and Chemical Biology, TU Dortmund University, Dortmund, Germany; 5 Center of Excellence, Physics of Life, Technische Universität Dresden, Dresden, Germany

**Keywords:** Intrinsically disordered proteins, Biochemistry, Chemical biology, Biophysical chemistry

## Abstract

It has long been proposed that phase-separated compartments can provide a basis for the formation of cellular precursors in prebiotic environments. However, we know very little about the properties of coacervates formed from simple peptides, their compatibility with ribozymes or their functional significance. Here we assess the conditions under which functional ribozymes form coacervates with simple peptides. We find coacervation to be most robust when transitioning from long homopeptides to shorter, more pre-biologically plausible heteropeptides. We mechanistically show that these RNA–peptide coacervates display peptide-dependent material properties and cofactor concentrations. We find that the interspacing of cationic and neutral amino acids increases RNA mobility, and we use isothermal calorimetry to reveal sequence-dependent Mg^2+^ partitioning, two critical factors that together enable ribozyme activity. Our results establish how peptides of limited length, homogeneity and charge density facilitate the compartmentalization of active ribozymes into non-gelating, magnesium-rich coacervates, a scenario that could be applicable to cellular precursors with peptide-dependent functional phenotypes.

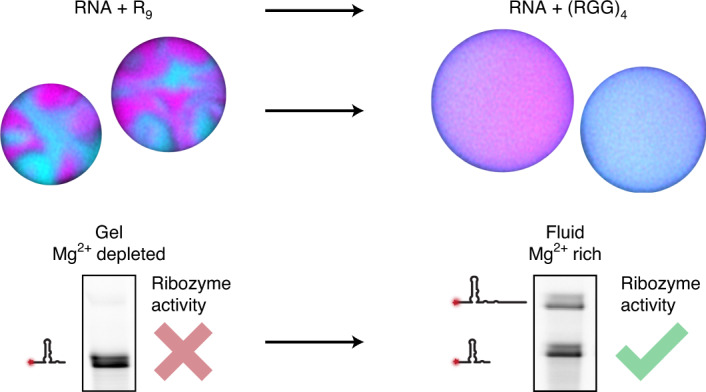

## Abstract (web summary)

Phase-separated compartments have long been proposed as precursors to cellular life. Now, it has been shown that RNA–peptide protocells are more robust when formed using shorter (rather than longer) peptides, and that peptide sequence determines the functional materials properties of these compartments.

## Main

The emergence of life on Earth has long been studied as a question of molecular reactions generating the building blocks of life such as peptides, lipids and nucleic acids^1^. Plausible pathways leading to complex polypeptides^2^ and ribozymes capable of the catalysis of elementary reactions such as RNA copying^3^, autocatalytic or cross-catalytic ligation^4^ and recombination^5,6^ are increasingly well understood. Beyond its chemical fingerprint, however, life is characterized by cellular organization. Therefore, an intriguing question is how molecular life could have transitioned into compartmentalized precursors of cellular life. In line with the classic view of biological cells as membrane-bound compartments, fatty acid vesicles have long been proposed as early cellular systems compatible with RNA encapsulation and activity^7^. More recently, however, it has become increasingly clear that liquid–liquid phase separation allows the formation of cellular compartments. Consequently, distinct biochemical environments with unique and dynamic properties and cellular functions can also be generated without the requirement for membranes^8,9^. Nevertheless, it remains unclear whether or how phase separation could have supported the transition from a molecular to a cellular world.

Complex coacervation has long been suggested as a plausible physicochemical process to explain pre-biological compartmentalization^10^. In particular, coacervate systems have the potential to achieve RNA compartmentalization via simple and widespread interaction of oppositely charged polyelectrolytes^11–14^. With the advent of phase separation in biology^9,15–19^, these systems have raised interesting possibilities for research into the origins of life^20–24^ and synthetic biology^25^. A number of studies have observed the formation of RNA-containing compartments from simple components^11,22,26^. Examples of complex coacervates include combining nucleic acids or nucleotides with positively charged polymers, such as spermine or spermidine^13^ and oligo- or polylysine^27^. Coacervates formed from long complementary RNA and polycationic peptides such as proline–arginine and proline–lysine repeats have been found to exhibit complex materials properties^26^. Here an apparently fluid peptide phase coexists with a static RNA gel, a phenomenon that has been described as the essentially decoupled dynamics of molecular components^26^. Furthermore, RNA biochemical reactions have been shown to proceed within liquid–liquid phase-separated compartments^21,25,28,29^, ribozymes in complex polyamine coacervates were found to allow RNA base pairing and template-directed polymerization^22^, and enhanced the activity of ribozyme cleavage reactions was found in artificial polydiallyldimethyl–ammonium/rA_11_ coacervates^22^. In a cellular context, condensates often include proteins and RNA^30^, as is the case for stress granules^31^, the nucleolus^32^ and germ granules^33^. Analysis of biological condensates suggests a molecular grammar based on protein sequence which could determine compartment function and properties^17,26,34^. In particular, reconstitution experiments with FUS protein revealed that different amino acids lead to different behaviour in RNA-free protein condensates. While arginine and tyrosine have been suggested to be important drivers of protein phase separation, the material properties of condensates are also determined by serine, glutamine and glycine residues^34^, the latter preventing ageing and solidification of protein droplets^34^. Finally, transitions in the material state of the cytoplasm and subcellular compartment have been suggested to control enzymatic activity, metabolic turnover and cell-cycle-dependent functionality^35–37^. For these versatile properties and potential functions of compartmentalization to extend origin-of-life scenarios, however, they would need to be compatible with peptides of plausible length and compositional purity, rather than highly evolved proteins or artificially long homopeptides.

In this article we study how phase-separated compartments could have formed during the origin of life. We find that the robustness of coacervation increases when transitioning from longer to shorter homopeptides. Weakening the constraints of peptide sequence further enables tuning of physiochemical properties and melting of RNA gels. Furthermore, we show that ribozyme function is modulated in a sequence-dependent manner, with peptide charge density as the major determinant of enzymatic activity. Mechanistic analysis reveals that coacervates form a distinct chemical environment in which peptide sequence determines concentrations of Mg^2+^ (an important cofactor of ribozyme reactions) without the need for a membrane. Our results establish how simple peptides can modulate the activity of ribozymes in plausible and evolutionarily attractive protocellular compartments, and emphasize opportunities to understand how biochemistry is regulated by the physicochemical properties of condensates in modern cells.

## Results

### Characterization of ribozyme–peptide coacervation regimes

**Fig. 1:**
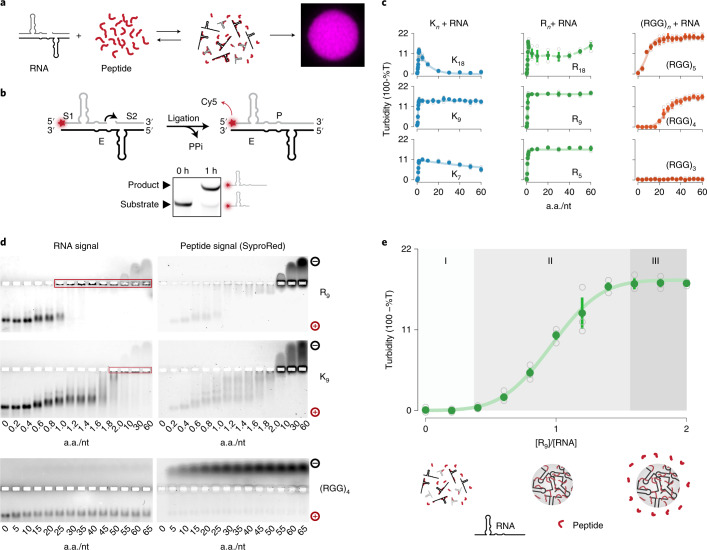
R3C ligase forms phase-separated coacervate compartments with simple cationic oligopeptides. **a**, Schematic representation of RNA–peptide compartment formation. The coacervate is shown as a fluorescent microscopy image with Cy5-labelled RNA. **b**, Schematic representation of the RNA-ligation reaction and experimental validation of R3C ligase product formation by gel electrophoresis. Reactions were carried out in 5 mM MgCl_2_ and 10 mM Tris pH 7.5 and contained 5 µM E, 0.1 µM Cy5-S1 and 6 µM S2. The band shift of Cy5 5′-labelled S1-RNA is indicative of ligation. **c**, Turbidity plots indicate the formation of stable coacervates over a broad peptide concentration regime for various R_*n*_ and longer (RGG)_*n*_ peptides, whereas for K_*n*_ peptides other than K_9_ coacervation was found to be concentration sensitive. Data are shown as mean ± 68% confidence interval; grey open circles indicate independent experiments; n = 3 for all peptides. %T describes the percentage of transmittance. All turbidity assays were performed using 5 µM E and 6 µM S2, a total of 438 µM of nucleotide. **d**, Electrophoretic mobility shift assays indicative of RNA–peptide complex formation using FAM-labelled RNA and SyproRed peptide staining. Samples contained 5 µM E, 6 µM S2 and 0.1 µM 6-FAM E in 5 mM MgCl_2_ and 10 mM Tris pH 7.5. Electrophoresis was carried at room temperature. Red boxes indicate accumulation of RNA in the wells. **e**, Turbidity plot for R_9_ showing a sigmoidal transition between soluble RNA–peptide complexes and coacervates. Data are shown as mean ± 68% confidence interval; grey open circles indicate independent experiments; n = 3. The plot shows three distinct regimes: soluble complexes (I), onset of coacervation (II) and appearance of peptide-saturated coacervates (III). Source data

Complex coacervation is known to occur via the phase separation of polyelectrolyte complexes^38,39^. To analyse the potential role of RNA–peptide coacervates in origin-of-life scenarios, we assessed the effect of charge and length of peptides on their formation (Fig. 1a). In particular, we focused on charge-interspaced heteropeptides in combination with catalytically active RNA molecules that promote RNA extension, such as the R3C ribozyme (E), which ligates two RNA substrates (S1 and S2)^4,40^ (Fig. 1b and Supplementary Materials).

**Extended Data Fig. 1:**
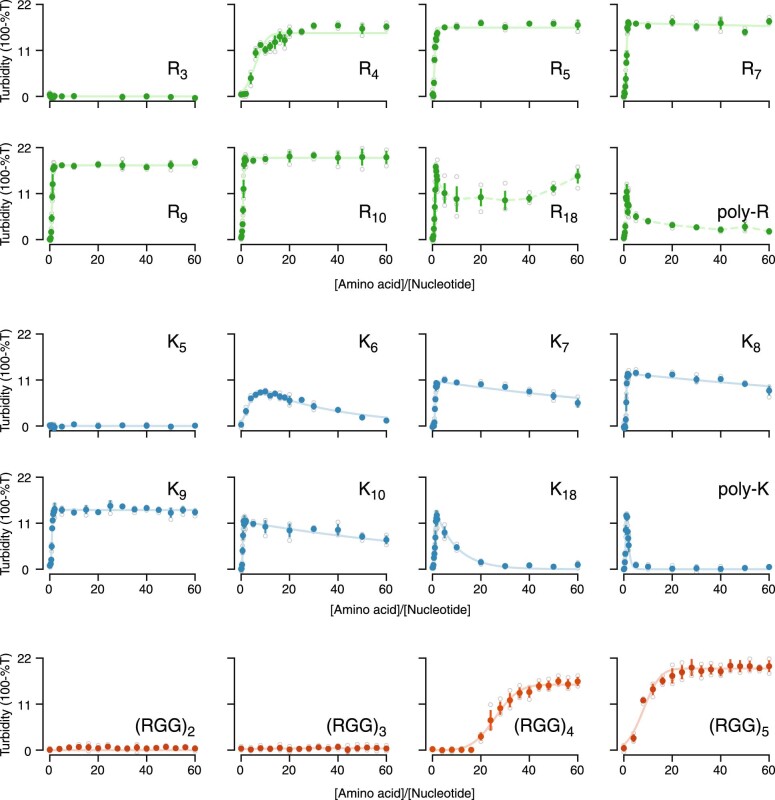
Arginine homopeptides and heteropeptides show greater stability under peptide excess. **a-c**, Sample turbidity at 500 nm of **(a)** R_n_, **(b)** K_n_ and **(c)** (RGG)_n_ peptides against amino acid-to-nucleotide ratio. Samples contained 5 µM E and 6 µM S2. At least 3 arginines, 6 lysines and 4 RGG repeats are necessary to achieve phase separation under standard reaction conditions. Lysine peptides different from K_9_ led to dissolution of the phase under peptide excess, most strikingly in the case of poly-K. R_n_ and (RGG)_n_ peptides formed stable phases. Data are shown as the mean ± 68% CI, open circles indicate individual experiments. Source data

The coacervation process was first studied through turbidity assays. Coacervates were prepared using 5 µM E and 6 µM S2 and varying concentrations of peptide in the presence of 10 mM Tris at a pH of 7.5 and 5 mM MgCl_2_, a minimal environment to support both coacervation and near-optimal ribozyme activity (Supplementary Fig. 1). Starting with homopolymeric peptide K_18_, we observed coacervation in turbidity assays at a charge ratio around unity (Fig. 1c). Increasing peptide concentration led to a reduction in turbidity and coacervate dissolution, leaving only a small range of amino acid/nucleotide (a.a./nt) ratios at which coacervates can form (Fig. 1c). To our surprise, when reducing the length of homolysine peptides further, we found that coacervates were still present at higher a.a./nt ratios, such that when using K_9_ (Fig. 1c), coacervates were observed from ratios of 0.5 to at least 60, implying a considerable robustness towards stoichiometric mismatches. Next, we asked if coacervation is specific to lysine or if other cationic peptides would also form coacervates together with the R3C ribozyme system. For arginine homopeptides (R_*n*_) (Fig. 1c), we found that an 18-residue arginine homopeptide formed coacervates at higher a.a./nt ratios than its homolysine counterpart. Furthermore, the ability to form coacervates at high a.a./nt ratios was maintained for R_9_ and shorter homoarginine peptides. This suggests that low-specificity charge-mediated interactions determine the coacervation process of short peptides with the R3C ligase. We thus conclude that the ability to form coacervates with ribozymal RNA is a generic property of cationic peptides with some differences in their robustness towards a.a./nt concentration mismatches. These differences between arginine and lysine homopeptides might arise from π–π stacking^41^ between arginine residues or arginine residues and RNA nucleotides, which renders arginine more suitable than lysine for generating coacervates. To our surprise, even arginine-rich heteropeptides formed from RGG repeats enabled robust coacervation when peptides were at least four repeats long (Fig. 1c and Extended Data Fig. 1).

### Charge neutralization drives coacervate formation

Next, we set out to better understand the mechanisms and sequence dependencies that govern RNA–peptide coacervate formation. To this end, we characterized complex formation of the R3C ligase system with the peptides R_9_, K_9_ and (RGG)_4_ by electrophoretic mobility shift assays (EMSAs) using non-denaturing agarose gels (Fig. 1d). In the case of R_9_, peptide concentrations below turbidity (a.a./nt ratio <1) caused only a mildly reduced electrophoretic mobility of RNA towards the anode with clear co-migration behaviour of the peptide. At peptide concentrations marking the onset of turbidity (a.a./nt ratio ≥1), RNA was unable to enter the gel matrix, suggesting a complete neutralization of the polyphosphate backbone. RNA mobility was ultimately reversed at a.a./nt ratios ≥2. A very similar trend was observed for K_9_. Intriguingly, for (RGG)_4_ both peptide and RNA mobility remained unaffected over the whole range of a.a./nt ratios and charge-neutral RNA–peptide complexes remaining inside the gel wells were not observed at any stoichiometry. This finding strongly suggests that RNA and (RGG)_4_ interact only weakly and their interaction is efficiently disrupted under EMSA conditions (1× Tris/Borate/EDTA buffer (TBE), *T* = 22 °C).

To extend our characterization of coacervate formation beyond turbidity assays, we employed transmission electron microscopy (TEM). We were particularly interested in observing whether there were soluble precursor complexes or free RNA molecules that coexisted with coacervates at the onset of turbidity. Furthermore, we wanted to check if the loss of solution turbidity was accompanied by dissolution of the coacervates. The TEM micrographs strengthened our previous model (Supplementary Figs. 2-4). At the onset of coacervation, we observed coacervates surrounded by filamentous structures similar to the ones observed under pure RNA conditions. The TEM micrographs also indicate coacervate dissolution for long homopeptides at high a.a./nt ratios.

Based on the above findings, we propose that three regimes of RNA–peptide self-assembly can be distinguished based on the a.a./nt ratio (Fig. 1e): a precursor regime (I), where RNA and peptides form negatively charged complexes that remain in solution; a transition regime (II), in which increasingly neutralized complexes phase separate; and a saturation regime (III), where coacervates form under an excess of positively charged peptides. The saturation regime can either span a broad peptide concentration range, as in the case of R_9_, or be limited to a relatively narrow range, as in the case of most lysine peptides. Taken together, these data indicate that ribozymal RNA phase separates with a wide range of cationic peptides. From these peptides, short and charge-interspaced peptides, despite weaker RNA binding, show the highest robustness towards coacervation with respect to stoichiometric charge mismatches. These findings show that peptide coacervation of ribozymes requires neither artificial and long homopeptides nor highly evolved proteins.

### (RGG)_4_-based coacervates sustain ribozyme activity

**Fig. 2:**
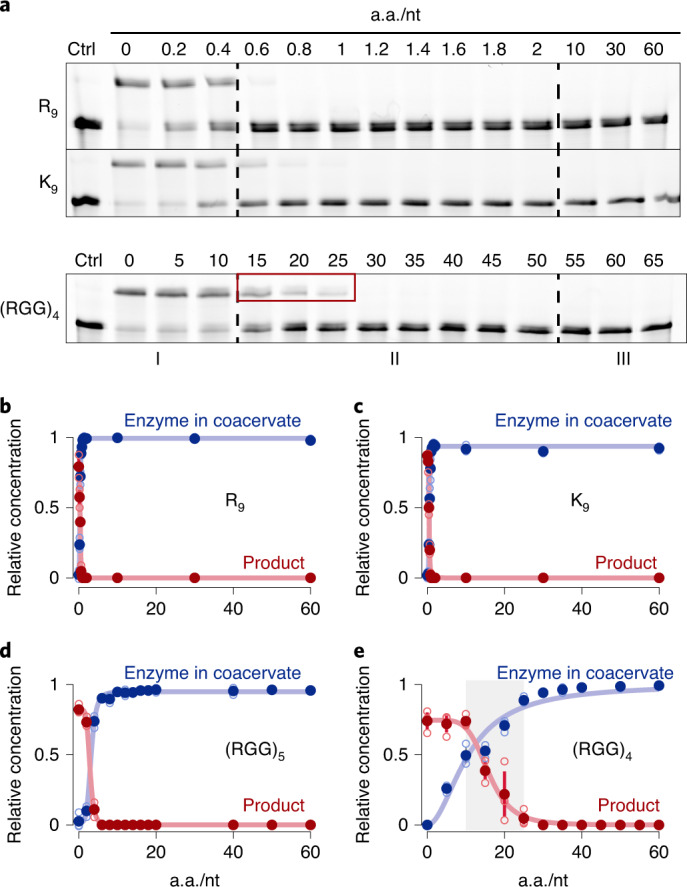
Ribozyme ligation activity is enhanced in coacervates containing charge-interspaced peptides as compared to homopeptides. **a**, 12.5% polyacrylamide urea denaturing gels show that ligation activity is maintained up to a ratio of 30 a.a./nt for (RGG)_4_. Product formation is rapidly lost for R_9_, K_9_ and (RGG)_5_ coacervates. Ctrl, control. The red box indicates residual enzymatic activity after the onset of coacervation. **b**–**e**, Relative amount of ligated substrate by gel analysis (red curve) and enzyme fraction (blue curve) in the coacervate phase against amino acid-to-nucleotide ratio for R_9_ (**b**), K_9_ (**c**), (RGG)_5_ (**d**) and (RGG)_4_ (**e**). Enzyme concentration in the coacervate phase was fitted to a Hill equation. Data are shown as mean ± 68% confidence interval; *n* = 3. Open blue circles indicate individual experiments. Product formation was fitted to a Hill equation. Data are shown as mean ± 68% confidence interval; *n* = 3. Open red circles indicate individual experiments. Source data

We next tested whether ribozyme ligase activity is sustained within the coacervate compartments. Recently, RNA enzymatic activity has been demonstrated in coacervates formed from poly-l-lysine/CM-dextran^21^ or polydiallyldimethylammonium (PDAC)/rA_11_ (ref. ^22^) systems that sequestered variants of the Hammerhead ribozyme. Here we wanted to test whether coacervates formed from simple peptides, RNA enzymes and their substrates at standard reaction concentrations can sustain enzymatic activity. To this end, we quantified fluorescently labelled RNA ligation products retrieved from coacervation assays at different a.a./nt ratios by gel electrophoresis. To quantify the amount of enzyme in the coacervates irrespective of their sizes, turbidity plots were complemented by sequestration measurements after separating spun-down coacervates and supernatant. All reactions contained 5 µM E, 6 µM S2 and 0.1 µM Cy5-S1 (see Fig. 1b and Supplementary Information for details). When R_*n*_ or K_*n*_ peptides were used, the onset of coacervation (entry into the transition regime) appeared to be correlated with a strong inhibition of the ligation reaction (Fig. 2a–c, Supplementary Figs. 5and 6), similar to previous observations using R_10_–rA_11_ coacervates with the Hammerhead ribozyme^22^. This indicates that pure R_*n*_– or K_*n*_–RNA coacervates are unlikely to be suitable host compartments for RNA-catalysed ligation reactions despite phase-separation-driven increases in concentration. In stark contrast, we observed RNA ligation in the presence of the (RGG)_4_ peptide at up to 20-fold a.a./nt excess (Fig. 2a,e and Supplementary Fig. 7). Although RNA does not completely reside within the coacervate phase under these conditions, this first observation indicates that ribozyme-based coacervate compartments can potentially host ribozyme reactions and that catalytic activity depends on peptide choice.

### Peptide composition determines mobility of the RNA phase

**Fig. 3:**
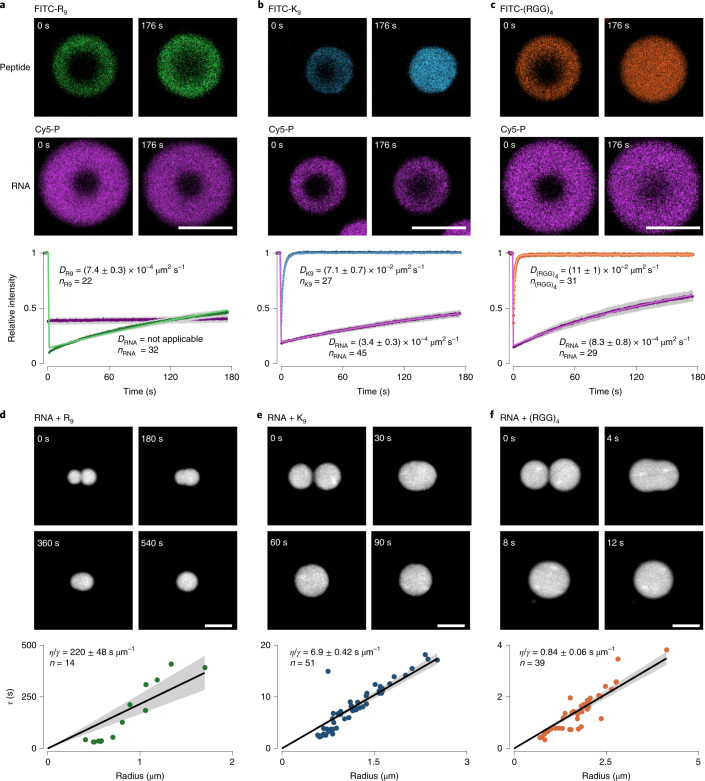
Peptide identity tunes mobility of RNA phase and resulting material properties of coacervates. **a**, With poly(R) peptides (here R_9_), ribozymes and their complementary products form a gel-like phase despite rapid peptide diffusion. **b**, Peptide-dependent mobility increase of previously gelling RNA for short polylysine peptides (K_9_). **c**, RNA-signal recovery after photobleaching indicates even faster diffusion and more complete recovery for (RGG)_4_ peptides. **d**–**f**, Droplet fusion experiments for RNA combined with R_9_ (**d**), K_9_ (**e**) and (RGG)_4_ (**f**) further indicate mechanical softening of RNA gel in a peptide-dependent manner. In all cases, the ribozyme ligation RNA product was labelled with Cy5. The peptides were labelled with FITC at the N terminus. The shaded region represents the 95% confidence interval of the mean of the points. Lower panels show the characteristic relaxation time of fusion for two droplets plotted against their geometric length. Data were fitted with a linear fit while constraining the *y* intercept to 0. The inverse capillary velocity corresponds to the slope of the fitted line and is defined as the ratio between viscosity (η) and surface tension (γ). The shaded region represents the 95% confidence interval of the fit. Scale bars, 5 µm. Source data

The differences in ribozyme activity observed in condensates containing (RGG)_4_ and the other peptides suggests locally distinct physicochemical properties that modulate ribozyme activity. To explore this further, we first analysed RNA and peptide mobility in coacervates by fluorescence recovery after photobleaching (FRAP)^42,43^. Coacervates were prepared at excess peptide, which is readily achieved at an a.a./nt ratio of 10 for R_9_ and K_9_, and an a.a./nt ratio of 70 for (RGG)_4_. When labelling R_9_ with fluorescein isothiocyanate (FITC), we observed rapid recovery of the fluorescence signal on a time scale of minutes, yet there was almost no mobility of Cy5-labelled-product RNA (labelling density <1% in both cases) (Fig. 3a). In agreement with the view of a rigid RNA phase, coacervates required multiple minutes for full coalescence, and quantitative analysis revealed an inverse capillary velocity as slow as 220 ± 48 s µm^−1^. These observations indicate that the RNA phase can be best described as gel-like.

This behaviour is similar to that of gels formed from highly complementary RNA, which coexist with freely diffusing peptides, resulting in two molecularly distinct phases that have been described as spatially overlapping but, in their dynamics, essentially decoupled^26^. In our case, RNA gelation was generally observed starting from R_18_ down to R_7_. Deviating from previous descriptions^26^, we observed this decoupling of molecular dynamics to break down when moving away from long and highly charged peptides, towards shorter, charge-interspaced peptides. The first indication for this was observed for coacervates made from RNA and R_5_, where some FRAP recovery of the RNA phase was noticeable after minutes (Supplementary Fig. 8). Furthermore, we found a trend of increased RNA recovery with lysine peptides of decreasing length (compare Fig. 3b with Supplementary Fig. 9). This recovery was clearly noticeable already at K_18_ and became progressively faster for peptides of decreasing length down to K_7_ (Supplementary Fig. 9). Thus, the choice of peptide has the potential to impact not only its own diffusion rate but also the mobility of the RNA phase. This peptide-dependent diffusivity of the RNA further accelerates when transitioning to charge-interspaced (RGG)_*n*_ peptides (Fig. 3b,c, and Supplementary Figs. 10 and 11). Likewise, here the droplet fusion was accelerated. K_9_ shows a >30-fold increase in fusion speed compared with R_9_-based coacervates. This fusion speed increases by almost another order of magnitude when transitioning to (RGG)_4_-based peptides (Fig. 3b,c,e,f). Taken together, our findings support a view in which short, charge-interspaced peptides melt RNA gels in coacervate phases, thereby enabling RNA mobility.

**Fig. 4:**
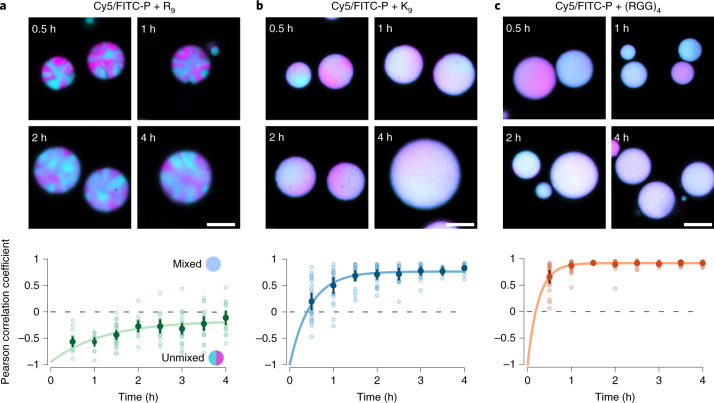
Peptide sequence determines RNA diffusion and subdomain formation in coacervate droplets. **a**–**c**, Fusion droplets of RNA with R_9_ (**a**), K_9_ (**b**) and (RGG)_4_ (**c**). Upper panels: confocal fluorescence microscopy images of droplets prepared with either Cy5- or FITC-labelled product RNA. Populations were mixed to allow fusion to occur and fused droplets (exhibiting both Cy5 and FITC signal) were monitored over time. R_9_ droplets (**a**) featured stable RNA domains after droplet fusion. RNA in K_9_ droplets (**b**) and (RGG)_4_ droplets (**c**) was generally well mixed. Scale bars, 5 µm. Lower panels: the observed change in the Pearson correlation coefficient between the two channels over time. The error bars show the 95% confidence interval of the mean. Data were fitted to a single exponential curve as a guide. Source data

Next, we asked if differences in RNA diffusion impact RNA distribution over longer time scales and if differential RNA gelation could be made visible. For this, we prepared droplets with differently labelled RNA and monitored RNA rearrangement after droplet fusion. Droplets were imaged every 30 min for up to 4 h after mixing (Fig. 4). For R_9_ droplets, we observed the formation of RNA subdomains that, despite being only micron-sized and displaying fusion on the time scale of a few minutes, persisted for the total experimental time, directly showing the presence of a gel-like phase (Fig. 4a). In contrast, K_9_ and (RGG)_*n*_ droplets did not maintain subdomains (Fig. 4b,c and Supplementary Fig. 11), and thus can be best described as viscous, liquid-like phases that enable unconstrained RNA motion leading to homogeneous distributions.

In summary, the data from these mobility analysis experiments suggest that coacervates formed from ribozymal RNA and short peptides can exhibit material properties ranging from a gel-like phase capable of maintaining subdomains for long periods of time to liquid-like phases of varying viscosity which permit the rapid homogeneous distribution of the components. As observed previously^26^, in the case of RNA gelation, the mobility of peptides and RNA can be drastically different. Importantly, in our regime of short peptides and RNA, the previously observed decoupling of RNA and peptide dynamics^26^ may be lost because RNA mobility becomes crucially dependent on peptide composition, which facilitates the melting of RNA gels. Furthermore, RNA mobility even shows amino-acid specificity, with arginine having the strongest impact on RNA diffusion. This is in line with arginine’s prominent role in peptide-based RNA aptamers^44^ and this high affinity may be sufficient to explain the absence of ribozyme activity in the gel phase.

### K_*n*_ and (RGG)_*n*_ coacervates do not disrupt RNA base pairing

**Fig. 5:**
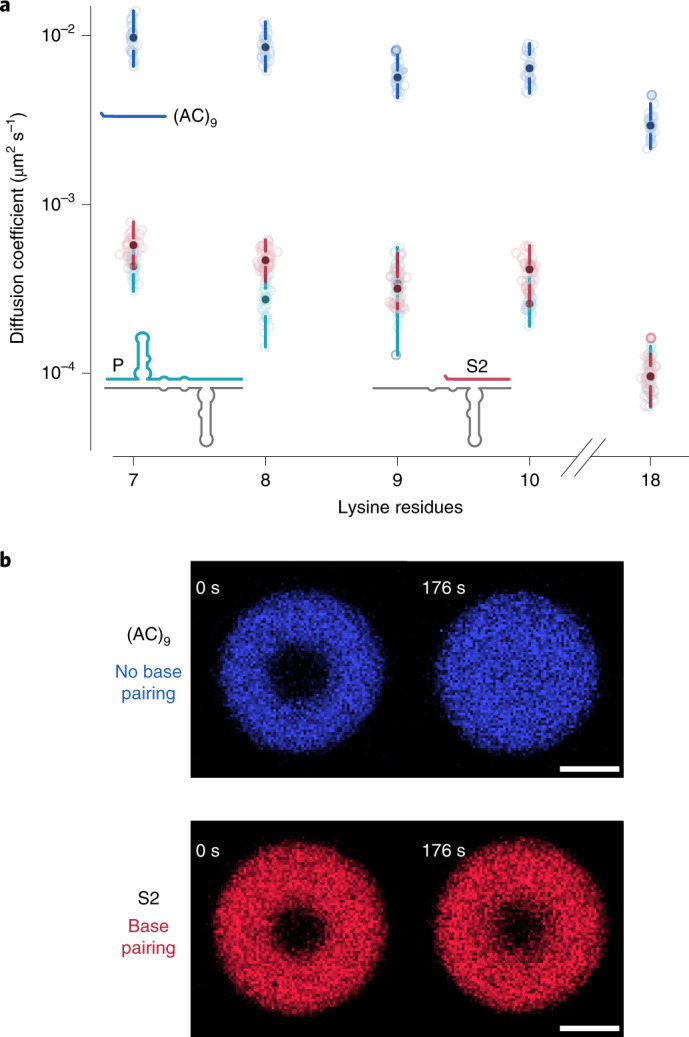
K_***n***_ coacervates permit RNA–RNA interactions. **a**, Boxplot of Cy5-P, Cy5-S2 and Cy5-(AC)_9_ apparent diffusion coefficients with filled circles representing the median, the space between the circles and the lines representing the second and third quartile, and the lines indicating the first and fourth quartiles. Scatter points represent independent observations. *n* for each experiment set is specified in Extended Data Fig. 2 and Supplementary Figs. 9 and 13. **b**, Representative images of (AC)_9_ and S2 RNA diffusion after FRAP in K_9_ coacervates. Scale bars, 5 µm. Source data

**Extended Data Fig. 2:**
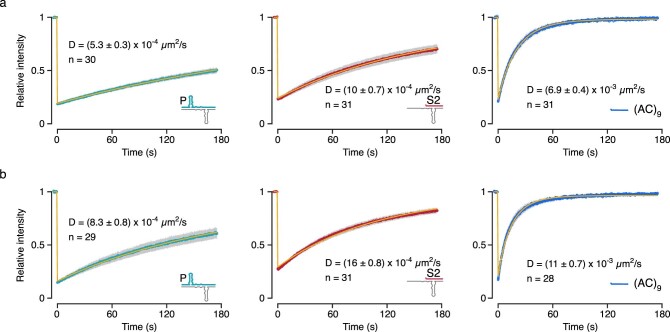
(RGG)_n_ coacervates permit RNA-RNA interactions. FRAP curves of Cy5-P, Cy5-S2 and Cy5-(AC)9 in (a) (RGG)5 and (b) (RGG)4 coacervates. Curves show that even though Cy5-S2 and Cy5(AC)9 RNAs have the same nucleotide number, their diffusion is markedly different. Indeed, Cy5-S2 diffusion is similar to Cy5-P diffusion, suggesting that both RNAs are able to interact with the longer E RNA. Apparent diffusion coefficients were derived from individual tracesCy5-S2 curves were forced to reach complete recovery, (AC)9 has a free parameter for maximal recovery. Data is shown as the mean of n individual determinations; shaded areas represent 95% CI intervals. Source data

Our data bring up the interesting question of which factors beyond the mechanical properties of the RNA phase may determine differential ribozyme activity in lysine-rich and RGG-based coacervates. One possibility is that RNA base pairing is disrupted inside coacervates, which could explain the absence of ribozyme activity in the fluid K_9_ coacervates. To address this, we used FRAP. We expected that stable complex formation should lead to similar diffusion coefficients for both the short RNA substrate and the ligation product (S2 and P), despite their 58-nt length difference, as they are part of the complexes E–P or E–S2 (Fig. 1b). However, in a hypothetical scenario of suppressed base pairing in coacervates, the diffusion of P and S2 should vary significantly due to their different hydrodynamic radii, and S2 should have a diffusion coefficient similar to that of a non-base-pairing substrate of the same length (control RNA (AC)_9_). Our data show that the apparent diffusion coefficients for the two RNA strands (P and S2) are very similar for K_*n*_ and (RGG)_*n*_ coacervates (Extended Data Fig. 2 and Supplementary Fig. 12), whereas the 18-nt non-complementary (AC)_9_ RNA oligomer exhibited between 5- and 30-fold higher diffusion coefficients (Fig. 5). These results suggest that base pairing takes place in both K_*n*_ and (RGG)_*n*_ coacervates and differences in ribozyme activity therefore cannot be explained by impaired RNA–RNA interactions.

### Peptide sequence determines coacervate magnesium content

**Fig. 6:**
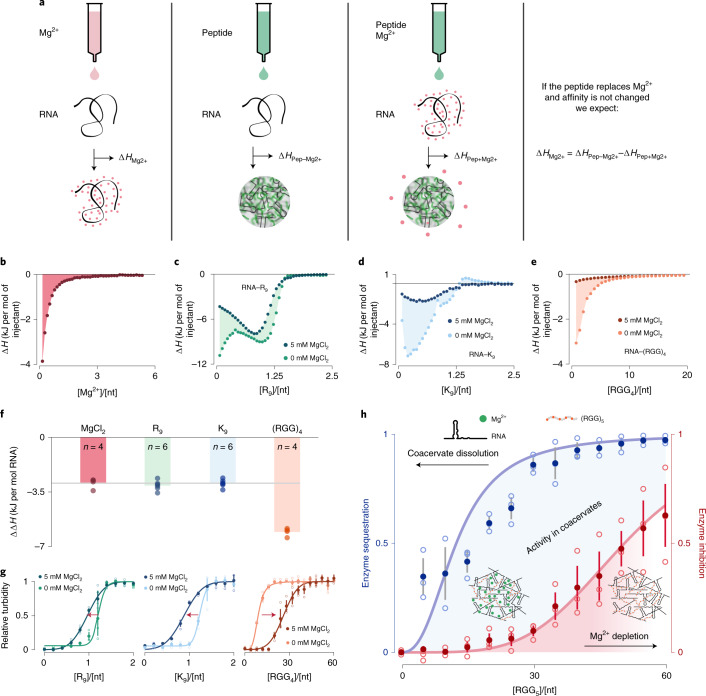
Isothermal titration calorimetry reveals sequence-dependent Mg^2+^ release from RNA upon peptide binding. **a**, Schematic representation of ITC experiments to measure the heat released upon Mg^2+^ binding to RNA (∆*H*_Mg_) and the heat produced upon peptide binding in the absence (∆*H*_Pep−Mg_) or presence (∆*H*_Pep+Mg_) of MgCl_2_. **b**, Injection heat curve of Mg^2+^ binding to torula yeast RNA. Each individual injection heat has been normalized by the amount of titrant injected and is plotted against the molar ratio of nucleotides over Mg^2+^ concentration in the cell. The total heat of the process corresponds to the sum of the injection heats, represented as the shaded area. **c**–**e**, Injection heat curves of R_9_ (**c**), K_9_ (**d**) and (RGG)_4_ (**e**). Each plot contains the injection curve of RNA–peptide binding in the presence or absence of Mg^2+^. The shaded area represents ∆*H*_Pep−Mg2+_ − ∆*H*_Pep+Mg2+_. **f**, Bar plot of ∆*H*_Mg2+_ and ∆*H*_Pep−__Mg2+_ − ∆*H*_Pep+Mg2+_ for R_9_, K_9_ and (RGG)_4_. **g**, Changes in turbidity plots caused by the presence of Mg^2+^. The presence of 5 mM MgCl_2_ enhances the coacervation process in R_9_ and K_9_ but moves the coacervation onset in (RGG)_4_ to higher amino acid ratios, indicative of Mg^2+^ competition with the peptide. **h**, Enzyme sequestration (blue curve) and enzyme inhibition (red curve) as a function of the a.a./nt ratio of (RGG)_5_ coacervates prepared at 20 mM MgCl_2_. The area between these two curves shows the a.a./nt ratio at which enzymatic activity can be found in the coacervate phase. Data are shown as mean ± 68% confidence interval; n = 3 for both curves. Open circles indicate independent experiments. Source data

**Extended Data Fig. 3:**
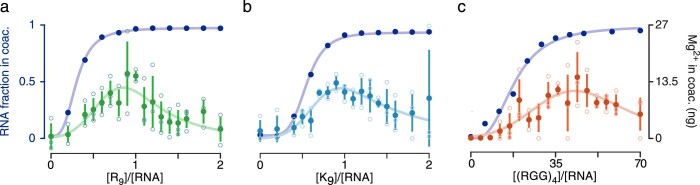
Ligation activity correlates with efficient Mg^2+^ sequestration into coacervates. **a-c** Magnesium mass content in the coacervate phase for (**a**) R_9_, (**b**) K_9_ and (**c**) (RGG)_4_ in nanograms. Mg^2+^ partitioning was measured by Inductively Coupled Plasma Mass Spectrometry (ICP-MS), n = 3; data are shown as the mean ± 68% CI, open circles indicate individual observations. Data points were fitted to a convolution of a gaussian with an exponential curve; for the fitting of the curve in (**b**), the last data point was not considered. Purple curves show an estimate of the total RNA fraction (E and S2) in the coacervate phase. Source data

Apart from molecular mobility and base pairing, ribozyme activity requires sufficient amounts of Mg^2+^ both for faithfully maintaining secondary RNA structure and as an essential cofactor for catalysis. Therefore, a differential presence of Mg^2+^ in coacervates could explain different levels of ribozyme activity, especially when comparing K_9_ and (RGG)_4_ coacervates. To specifically test the effect of peptide choice on the presence of magnesium in our coacervates, we used two complementary techniques: inductively coupled plasma mass spectrometry (ICP-MS) for determination of magnesium in the coacervate phase (Extended Data Fig. 3) and isothermal titration calorimetry (ITC) to assess magnesium-induced differences in the coacervation process (Fig. 6). The ICP-MS data indicated that peptide excess causes Mg^2+^ depletion from the coacervate phase for R_9_, K_9_ and (RGG)_4_ compartments (Extended Data Fig. 3). For all coacervates tested, Mg^2+^ was depleted under peptide excess; however, its depletion efficiency depended on the peptide species. R_9_ and K_9_ coacervates showed almost complete Mg^2+^ release at an a.a./nt ratio of 2, whereas (RGG)_4_ coacervates required drastically increased a.a./nt ratios of >70 for full Mg^2+^ release.

To study the process of magnesium depletion in more detail, with higher resolution and largely independent of sequence biases, we carried out systematic ITC experiments using increased quantities of RNA (torula yeast RNA, Sigma). We observed that Mg^2+^ binding to the RNA is an exothermic process^45^ with a total heat release $\Delta{H}_{\mathrm{Mg}}=-2.9\pm0.2\,{\mathrm{kJ}}\,{\mathrm{mol}}_{\mathrm{DNA}}^{-1}$ (mean ± 68% confidence interval) (Fig. 6a,b,f). We reasoned that, when comparing RNA coacervation with peptides in the presence and absence of Mg^2+^, the total enthalpic difference ∆∆*H* should equal ∆*H*_Mg_ if Mg^2+^ is completely released from the RNA (Fig. 6a). In other words, if the peptide were to replace the magnesium, then the heat release upon peptide–RNA binding should be reduced by the amount of heat that the Mg^2+^ released when previously binding to the RNA. We therefore measured the heat released upon peptide binding to the RNA alone (∆*H*_Pep−Mg_) and to RNA preincubated (∆*H*_Pep+Mg_) with 5 mM MgCl_2_ (Fig. 6c–e). In agreement with the coarser mass-spectrometry data, we found that heat release was observed with R_9_ and K_9_ addition up to an a.a./nt ratio of approximately 1.25.

When comparing the heat release upon R_9_ and K_9_ titration in the absence and presence of Mg^2+^, we found that the ∆∆*H* of peptide binding to the RNA had an enthalpic cost precisely equal to the heat release of Mg^2+^ binding to R_9_ and K_9_ (Fig. 6f). This indicates that R_9_ and K_9_ competitively replace all the magnesium from the coacervates, a process that is largely terminated already for a small fractional excess of peptides. These data suggest that K_9_ and R_9_ bind the RNA with high affinity, and that the resulting absence of Mg^2+^ explains the lack of ribozyme activity in these coacervates, especially in fluid K_9_ coacervates. In line with this view, we found that the chosen R3C ligase was inhibited at Mg^2+^ levels below 1 mM (Supplementary Fig. 1). When studying coacervation of RNA with (RGG)_4_, on the other hand, we observed heat release over a wide concentration range far beyond a precise stoichiometric ratio, with saturation only from an approximately 10-fold excess of amino acids (Fig. 6e, note the different dimensions of the *x* axis). Furthermore, we observed a highly differential response for (RGG)_4_ binding to RNA with Mg^2+^ incubation. In particular, we observed a highly reduced heat release when the negatively charged RNA was already neutralized by Mg^2+^ counter-ions, in which case the ∆∆*H* even exceeded the penalty of Mg^2+^ replacement (Fig. 6e). Over a wide concentration range, heat release was strongly reduced compared to what one would expect for (RGG)_4_ binding to RNA, even when considering the additional penalty of replacing magnesium. Thermodynamically, this rules out firm binding of (RGG)_4_ to RNA in the presence of magnesium. This lasting presence of RNA-bound Mg^2+^ in (RGG)_4_-based coacervates probably explains the maintenance of ribozyme activity.

To understand this result, it is necessary to consider that Mg^2+^ is able to efficiently compete with (RGG)_4_ for RNA binding, thus effectively changing the equilibrium constant of the process. In general, the efficiency of such competition depends on the affinity of the peptides for the RNA. Hence, we predict that if the equilibrium of cationic ion binding to RNA is shifted from peptides to Mg^2+^ in a sequence-dependent manner, we should see differential effects of Mg^2+^ presence on the coacervation process, for example, when replacing R_9_/K_9_ with (RGG)_4_. We therefore proceeded to investigate the effect of 5 mM MgCl_2_ on the coacervation process to address the effect of Mg^2+^ on the formation of (RGG)_4_ coacervates.

For this purpose, we complemented the previously shown turbidity assays with their equivalent in the absence of MgCl_2_. As expected from the ITC data, MgCl_2_ promotes coacervation with R_9_ and K_9_, seen as a left shift of the turbidity curves (Fig. 6g). Here the Mg^2+^ mainly helps to achieve charge neutrality, as explained later. In stark contrast, the presence of MgCl_2_ meant that larger amounts of (RGG)_4_ peptide were required to form coacervates, seen as a right shift of the turbidity curve (Fig. 6g). In particular at intermediate (RGG)_4_ concentrations, these data directly show that magnesium is capable of blocking essential interactions between a weak-binding peptide and the RNA, such that coacervate dissolution is observed. Similarly, this shielding effect of magnesium can also be observed with (KGG)_7_ (Supplementary Fig. 13), suggesting that this effect is determined by the interspacing of positive charges rather than the precise choice of the cationic amino acid (Supplementary Fig. 13).

### Regimes of peptide-dependent functional material properties

**Extended Data Fig. 4:**
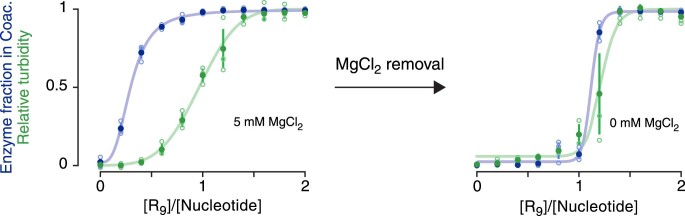
RNA partitioning depends on Mg^2+^. The purple curves show the enzyme RNA fraction in the coacervate phase against a.a./nt. Coacervates were prepared using 5 mM MgCl_2_ (left panel) or 0 mM MgCl_2_ (right panel). Filled circles show the mean of three independent experiments with a 68% confidence interval, open circles indicate individual observations. The partitioning data was fitted to a Hill equation and the turbidity data to a sigma function. Source data

Interestingly, the width of the transition regime (regime II, see Fig. 1) increases in the presence of MgCl_2_ (Fig. 6g). Comparison of the turbidity plots for R_9_ with partitioning curves at 0 and 5 mM MgCl_2_ revealed that RNA partitioning is enhanced by the presence of MgCl_2_ (Extended Data Fig. 4). In the absence of Mg^2+^, the turbidity and partitioning curves closely resemble a step function, and both turbidity and partitioning onset are observed at an a.a./nt ratio close to unity (Extended Data Fig. 4). This indicates that coacervates only form once the RNA has been completely neutralized by the peptide. Overall, this suggests a partial neutralization of RNA by Mg^2+^ ions, which subsequently allows for the formation of coacervates at a.a./nt charge ratios below unity. This widening effect is most pronounced for the tightly binding R_9_ and K_9_ peptides, but it can still be observed for (RGG)_4_, although coacervation here requires high peptide levels due the RNA-shielding capacity of the Mg^2+^ ions.

Our systematic analysis reveals that the phenomenologically determined regimes in turbidity plots correspond to three functionally distinct regimes in which ribozyme activity is linked to the physicochemical properties of coacervates (Fig. 1e). In the first region (I), when there is no turbidity present, only complexes exist and ribozyme activity is observed in solution. In the second region (II), full RNA partitioning is achieved by partial neutralization of the RNA by Mg^2+^. This region permits ribozyme activity given that the peptide-dependent material state is sufficiently fluid. Finally, in region (III), an excess of cationic peptide competitively depletes Mg^2+^ from coacervates, which accounts for a loss of ribozyme activity.

### Tuning RNA–peptide affinity recovers RNA enzymatic activity

Our results indicate that RNA–peptide interactions displace Mg^2+^ ions and that weaker interactions should lead to higher Mg^2+^ concentrations in the coacervate phase. We therefore reasoned that RNA–peptide affinity can be tuned by the use of different MgCl_2_ concentrations in the system. Thus, we hypothesized that, if our observations hold true, reducing the RNA peptide affinity in (RGG)_5_ coacervates by increasing MgCl_2_ concentration from 5 mM to 20 mM would lead to a recovery of enzymatic activity. As expected, under these conditions, RNA enzymatic activity was maintained even after close to total enzyme RNA sequestration in the coacervate phase (Fig. 6h and Supplementary Fig. 14). Furthermore, we found low RNA in-and-out diffusion in these coacervates, which excludes the possibility that the ligation reactions will take place in the solution, and which can probably be explained by diffusion-limiting RNA interactions at 20 mM Mg^2+^ concentrations (Supplementary Figs. 15 and 16 and Supplementary Information).

To further generalize our observations, we also tested if charge interspacing would have similar effects on coacervates based on lysine-rich peptides and RNA. When transitioning from K_9_ to (KGG)_9_, we observed both a 120-fold acceleration in droplet fusion times (Supplementary Fig. 17) and a characteristic right shift of the turbidity curve upon Mg^2+^ increase, indicating the competitive replacement of peptides by Mg^2+^. Consequently, enzymatic activity is also present in these coacervates at 20 mM Mg^2+^ (Supplementary Fig. 17). These results indicate that charge interspacing is a general physicochemical mechanism modulating the material properties of RNA–peptide coacervates.

## Discussion

While protocells are frequently understood as membrane-bound compartments, our results show how simple and plausible heterogeneous peptides are capable of forming functionally diverse compartments and suggest that such coacervates could be considered an attractive alternative in origin-of-life scenarios. In particular, we find that loosening chemical constraints on peptide composition increases the plausibility of coacervate formation and provides access to compartments with sequence-dependent, functional material properties. Conceptually, our results indicate how peptides within reach of prebiotic synthesis could have facilitated the early transition of a putative RNA world to an evolutionarily compelling scenario of protocellular compartments with sequence-dependent phenotypes. Furthermore, our methodology might provide an interesting platform to understand regulation in modern cells.

### Plausibility

Our study showed that coacervate formation with simple ribozymes requires neither proteins nor artificially long homopeptides. In particular, systematic analysis reveals that shorter peptides may even increase the robustness of protocellular compartmentalization because they provide stability for coacervates at high peptide ratios (regimes II–III). Of particular interest for origin-of-life scenarios, we find that charge-interspaced heteropeptides, which are statistically and chemically more plausible, have the highest potential to maintain the activity of ribozymes compared with arginine and lysine homopeptides.

In the absence of a genetic code to determine peptide sequence, charge interspacing by glycine has two distinct implications for plausibility. (1) Moving away from artificial homopolymers towards heteropolymer boosts the combinatorial plausibility of a pre-biological scenario in which long homopolymers were likely to be scarce. As an example, the combinatorial likelihood of generating a 12-a.a. heteropeptide containing 66% glycine (that is, 8G and 4R), would have been more than 10,000 times (= 12 × 11 × 10 × 9) that of an equally long homopeptide, and adding only three more residues would have increased this factor to >360,000. Importantly, these probabilities increase exponentially with the numbers of peptide molecules required to form a coacervate. (2) Additionally, the much simpler glycine further boosts the chemical plausibly of interspaced and only weakly charged peptides because cationic peptides, including arginine and even the non-canonical but simpler ornithine, were likely to have a lower abundance among pre-biological amino acids^46^. Notably, we observed similar behaviour for charge-interspaced lysine peptides, stressing the universality of these findings. Thus, considering stability and plausibility arguments, we propose charge-interspaced peptides as an evolutionarily relevant motif for the transition from molecular to coacervate precursors of life.

### Evolutionary attractiveness

While we can only speculate how cells evolved, it is clear that at some stage RNA and peptides, two chemical classes of central importance for cellular life, must have found themselves together in small compartments. When combining peptides within reach of pre-biological synthesis and model ribozymes, we find that coacervates, a long-standing suggestion for protocellular life, emerge. These not only show increased stability when increasing chemical and combinatorial plausibility, but we find emergent properties to be tunable in a peptide-dependent manner. Functionally, the reduced charge density of short heteropeptides influences the coacervate phase in several ways. The lower affinity of peptides such as (RGG)_*n*_ towards RNA causes a substantial widening of the transition regime (region II), which drastically extends the range of peptide concentration in which ribozymes maintain their activity. Mechanistically, the reduced charge density promotes magnesium partitioning and compartment fluidity, both described here as prerequisites for ribozyme activity inside coacervate compartments. More specifically, (1) our ITC data systematically show that peptide composition, a primitive constraint on sequence, is itself sufficient to generate locally distinct physicochemical conditions that modulate ribozyme function via differential metal ion concentrations without the need for a membrane^47^. (2) In addition to chemical identity, the physical conditions are highly sequence dependent. In particular, RNA base pairing has recently been described to lead to RNA gelation in coacervates and can still be observed for oligo-arginine below 10 residues. Interestingly, this absence of RNA mobility can be prevented by the charge interspacing of short peptides, which melt the RNA gel and promote ribozyme activity. Our results therefore establish how self-assembly drives the formation of protocellular compartments in which peptide sequence controls nucleic acid activity via the emergent physicochemical phenotypes. These findings represent a functional link between two important classes of molecules and provide a cellular basis upon which future selection processes could act.

### Analogies with regulation in modern cells

Interestingly, our results suggest that, as in bacteria and eukaryotic cells^35–37^, the earliest cellular metabolic processes (that is, RNA fixation via ligases) could have been tuned via the material properties of the compartment. In more evolved protocells, coexisting coacervate phases^26^ could have served different purposes, similar to spatial and temporal variations in the functional material properties of modern cells^35–37^. While gel-like droplets such as R_*n*_–RNA structures could function as RNA storage units that increased RNA stability, (RGG)_*n*_–RNA-like compartments could have formed with active RNA enzymes. Notably, RGG/RG motifs are also highly enriched in RNA-binding proteins in eukaryotes^48^ and mediate protein–RNA interactions^49–51^. Several proteins with RGG domains have been observed to phase separate in the presence of RNA, including LAF-1^52^, FUS^34,53,54^, PGL3^33^ and FIB1^16^. Our results suggest that the previously proposed fluidization of these condensates by glycine incorporation in mammalian proteins^34^ might also have relevance for other molecular species. This may include RNA mobility, and could even impact concentrations of co-factors such as small ions, which determine the biochemical identity of compartments. Given the low complexity of the molecular constituents required, charge interspacing might be an ancient evolutionary mechanism to locally modulate function in a sequence-dependent manner. The minimal models described here may further help to understand how amino acid changes alone suffice to modulate the material properties of RNA–protein biological condensates, while excluding the possibility of non-specific side effects that may result from amino-acid substitution in in vivo proteins.

To summarize, our results provide a mechanistic explanation for how simple heteropeptides maintain ribozyme activity in coacervates and provide a compelling proposition for the origin of cellular organization with rich and sequence-dependent functional phenotypes.

## Methods

### RNA in vitro transcription

E and S2 RNA strands were synthesized by in vitro transcription using a template DNA containing the T7 promoter in the 3′ region. The template DNA was hybridized with a 22-base complementary DNA strand containing the T7 promoter. Hybridization was carried out by mixing equimolar concentrations of template DNA and the T7 promoter complementary sequence. The hybridization mix was incubated in a water bath at ∼80 °C and left to cool to room temperature. This partially double-stranded DNA was then used for in vitro transcription. The in vitro transcription reaction contained the following: 20 mM MgCl_2_, 2 mM spermidine, 40 mM Tris–HCl pH 7.8, 3.75 mM nucleoside triphosphates (each nucleotide), 0.5 μM double-stranded DNA, 10 mM dithiothreitol, 0.09 μg μl^−1^ T7 RNA polymerase and 5 units ml^−1^ inorganic pyrophosphatase. The reaction was incubated for 1 h at 37 °C. After transcription, reactions were concentrated and buffer exchanged with water using Amicon 10 kDa molecular weight cut-off (MWCO) filters. Concentrated samples were then mixed with 2× loading buffer (95% formamide, 18 mM EDTA, 0.025% SDS) and heated to 80 °C for 2 min. E and S2 RNAs were run on 12.5% or 20% denaturing polyacrylamide gels (8 M urea, 1× TBE), respectively, at a constant 30 V cm^−1^. RNA bands were visualized by ultraviolet shadowing using a fluorescent thin-layer chromatography plate and exciting with 254 nm light. Bands of interest were excised from the gel and electroeluted for 30 min using Bio-Rad model 422 with 10 kDa MWCO filters. After electroelution, samples were recovered, filtered with Costar 0.22 μm cellulose acetate filters and buffer exchanged four times with RNase-free water (Ambion) using Amicon 10 kDa MWCO filters. Final RNA concentration was measured using a NanoDrop 2000 spectrophotometer (ThermoFisher) at 260 and 280 nm wavelengths and the respective molar extinction coefficient (*ε*) for each RNA strand (calculated using the IDT calculator).

### Turbidity assays

Samples contained 5 μM E, 6 μM S2, 10 mM Tris pH 7.5 and 5 mM MgCl_2_. Samples were prepared by addition of a master mix containing the RNA and buffer to a peptide solution. The final sample volume was 5 μl. After addition of RNA, samples were mixed for 20 s and incubated for a further 40 s. Then, 1.5 μL of sample was analysed using a NanoDrop 2000 with ultraviolet–visible settings. We measured absorbance at 500, 260 and 280 nm. Absorbance at 500 nm was then converted to turbidity using the following equation:

$${\mathrm{turbidity}} = 100 - 10^{(2 - {\mathrm{absorbance}})}$$

Turbidity curves against the a.a./nt ratio were fitted to the following equation to capture the sigmoidal increase in turbidity and the exponential decrease in turbidity for the K_*n*_ peptides, fitted with a function of a convolution of a Gaussian with an exponential curve:

$$\begin{array}{l}f\left( x \right) = \sigma \times A_1 \times \exp \left( {\frac{{ - x - x_0}}{\tau }} \right) \times \exp \left( \left( {\frac{{\sigma ^2}}{{4 \times \tau ^2}}} \right)\right.\\\qquad\quad\left. \times \left( {1 + {{{\mathrm{erf}}}}\left( {\left( {\frac{{x - x_0}}{\sigma }} \right) - \left( {\frac{\sigma }{{2 \times \tau }}} \right)} \right)} \right) \right)\end{array}$$

where *A*_1_ is a scaling factor, *σ* is the width of the Gaussian, *x*_0_ is the inflection point in the sigmoidal part of the turbidity curve and *τ* is the exponential decay constant.

### Fluorescence recovery after photobleaching and diffusion calculation

Coacervates were prepared by mixing a solution containing buffer, E, 6 μM S2 and 0.1 μM of the fluorescent RNA or 3 μM in monomer units of FAM-labelled peptide. When product diffusion was measured, the solution was incubated at room temperature for 30 min to allow ligation of S2 and Cy5-S1 by the enzyme. After mixing with the peptide solution, samples were incubated at room temperature for 1 min. After incubation, 5 μl of Pico-Surf was added to form a water–oil emulsion. Samples were then loaded in flow chambers prepared for imaging. Imaging was carried out using a Plan-Apochromat 63×/1.4 oil DIC objective and a Zeiss LSM 880 Airy inverted confocal microscope. Images were taken every 200 ms for a total time of 3 min.

The mean intensity of four regions of interest—bleaching region, whole droplet, reference droplet and background—was collected for every frame. After image acquisition, videos were analysed using a custom-made MATLAB application with droplet tracking.

Images were normalized by first subtracting the background from the bleaching region, the whole droplet and the reference droplet. The fluorescence for the bleaching region and the whole droplet was then corrected for photobleaching by dividing by the reference droplet and normalized to the mean of the frames before bleaching (20 frames). The bleaching region intensity for every frame was then divided by the intensity of the whole droplet. This final curve was then fitted to an exponential equation:

$$f\left( t \right) = \left\{ {\begin{array}{*{20}{c}} {1,\,{\mathrm{for}}\;t < t_0 = 0\;s} \\ {1 - A \times \exp \left( {\frac{{ - t - t_{\mathrm{bleach}}}}{\tau }} \right) + C,\,x \ge 0} \end{array}} \right.$$

where t is the time and C the offset to complete recovery. For the cases in which full recovery was not observed, *C* was assumed to be 0. The fitted fluorescence recovery time constant (*τ*) was then used to calculate the diffusion coefficient *D* using the following experimental equation:

$$D = \frac{{0.88r^2}}{{4\tau \ln 2}}$$

where *r* is the radius of the bleaching region. To calculate a final diffusion coefficient for each coacervate condition, individual diffusion coefficients were averaged. This mean diffusion is reported with a 95% confidence interval.

### Torula yeast RNA purification

Torula yeast RNA was purified by first dissolving the RNA in MilliQ water. An equal volume of phenol:chloroform:isoamyl alcohol (25:24:1) was added to the RNA solution. The two-phase solution was vortexed to generate an emulsion and later centrifuged at 15,000 r.c.f. for 15 min. The aqueous phase was recovered and the process was repeated several times until no precipitate was observed between the aqueous and the organic phases. The final aqueous phase was taken and 3 M sodium acetate solution, preadjusted to pH 5.5, was added to a final concentration of 10% (v/v) To this solution, the same volume of isopropanol was added to precipitate the RNA. The pellet was finally washed twice with 70% ethanol. The washed pellet was then dissolved in MilliQ water. The final concentration of RNA was calculated using a NanoDrop 2000 and an average extinction coefficient for the nucleotides^55^ of *ε* = 8.9 × 10^−3^ μM^−1^ cm^−1^.

### Electrophoretic mobility shift assays

Samples contained 5 μM E, 6 μM S2 and 0.1 μM 6-FAM-E and were prepared in the same way as for the turbidity assays. After 1 min of incubation, 1.25 μl of 5× loading buffer (50% glycerol, 5× TBE, Orange G) was added to the samples. A 5 μl portion of each sample was then loaded in a 4% agarose gel. The gel was run at 6 V cm^−1^ for 20 min in 1× TBE buffer (89 mM Tris, 89 mM boric acid, 20 mM EDTA). After electrophoresis, 6-FAM fluorescence was collected using a Typhoon 9900 scanner. Gels were then incubated for 30 min in 0.05% SDS and stained for 30 min with SyproRed in 10% acetic acid. The gel was finally washed with 10% acetic acid for 5 min and then imaged to collect the SyproRed signal.

### Electron microscopy grid preparation and imaging

Carbon-coated grids (400 hexagonal mesh, copper, EMS) were prepared by depositing a thin carbon layer over the grids. The layer was obtained by carbon evaporation (Bal-Tec Med 020, Leica) onto muscovite (Mica Sheets, EMS). Grids were arranged on a mask and then placed on a homemade cuvette filled with distilled water. The carbon-coated muscovite was submerged in water to peel off the carbon layer and deposited on the grids by decreasing the water level of the cuvette. Grids were dried overnight and glow discharged before use (Bal-Tec Med 020, Leica). Samples for negative staining were prepared as for turbidity assays. After 1 min incubation, 5 μl was loaded on the grid. After 30 s, liquid was removed from the samples using Whatmann filter paper and samples were then immediately overlaid with 2% uranyl formate solution for another 30 s. After removing the uranyl formate, the grids were allowed to dry at room temperature. Imaging was performed using a Tecnai F30 transmission electron microscope.

### Coacervate RNA partitioning

Samples contained 5 μM E, 6 μM S2, 0.1 μM of FAM-labelled RNA to be tested, 10 mM Tris 7.5, 5 mM MgCl_2_ and peptide. Final sample volume was 5 μl. After mixing the RNA with the peptide, samples were centrifuged for 10 min at 20,000 r.c.f. After centrifugation, all the supernatant phase was transferred to a new tube. We then added 5 μl of 1× buffer to the tubes containing the coacervate phase. A 40 μl portion of 5 M NaCl was added to both the supernatant and coacervate. Finally, 12 μl was loaded in a 384-well plate with a flat plastic bottom, and each sample was measured in triplicate. The well plate was read on a TECAN Spark 20 M spectrophotometer using *λ*_exc_ = 492 nm and *λ*_em_ = 523 nm with a bandwidth of 10 nm at 25 °C.

To measure the partitioning of E for (RGG)_4_ and (RGG)_5_ and to exclude contamination by other shorter sequences produced during RNA chemical synthesis by the supplier, we prepared coacervates as stated before but centrifuged them for 2 min and analysed them by gel electrophoresis using 0.1 µM of FITC-labelled E RNA.

After data collection, we took the average fluorescence intensity of the triplicates and subtracted a reference background (containing buffer and NaCl). The intensity of the coacervate fraction was divided by the supernatant intensity. Data were then fitted to a Hill equation:

$$f\left( x \right) = F_{\mathrm{min}} + \frac{{F_{\mathrm{max}} - F_{\mathrm{min}}}}{{1 + \left( {\frac{{F_{\mathrm{half}}}}{x}} \right)^n}}$$

where *F*_max_ is the maximum fluorescence intensity in the coacervates phase, *F*_min_ is the minimum fluorescence intensity (typically close to 0 for no peptide), *F*_half_ is the peptide concentration at which half of the fluorescent RNA is retained in the coacervate phase and *n* is the Hill coefficient. For Fig. 6h, the data points were weighted by the reciprocal of the standard deviation and *F*_min_ constrained to 0.

### RNA ligation activity

Reactions were prepared by mixing a solution of E, S1, Cy5-S2 and Tris 7.5 with a solution of peptide and MgCl_2_ to yield final concentrations of 5 µM E, 6 µM S2, 0.1 µM Cy5-S1, 10 mM Tris 7.5, 5 mM MgCl_2_ and the respective concentrations of peptide depending on the a.a./nt ratio tested. The final volume was adjusted to 5 µl. Reactions were incubated for 1 h at 30 °C. Then, incubation reactions were stopped and coacervates dissolved by adding 4 µl of loading buffer (5.33 M guanidinium thiocyanate, 8 M urea) per 1 µl of sample. Samples were loaded on a 12.5% polyacrylamide gel (19:1) with 8 M urea and 1× TBE. Gels were ran at 10 V cm^−1^ for 40 min and imaged in a Typhoon 9900 scanner to visualize Cy5 fluorescence. After imaging, gels were analysed with a custom-made MATLAB script to subtract the background and calculate the fraction of Cy5-S1 ligated into product P.

### Droplet fusion

Droplet fusion experiments were performed in samples containing 5 µM E, 6 µM S2, 0.1 µM Cy5-S1 in 10 mM Tris 7.5 and 5 mM MgCl_2_. Droplets were prepared at an a.a./nt ratio of 10 for R_9_ and K_9_ coacervates, 40 for (RGG)_5_ and 70 for (RGG)_4_. Samples had a final volume of 5 µl. After coacervate preparation, 5 µl of Pico-Surf was added to encapsulate coacervates in a water–oil emulsion. Coacervates were imaged using a 60× UPLSAPO/1.2 water objective with W-IR coating (Olympus) mounted on a spinning disc confocal microscope. Cy5 was excited at *λ* = 633 nm with a Cy5 filter set. The microscope was equipped with a FLUCS setup^56^ that was used to bring droplets closer to each other.

Images were analysed using a custom-made MATLAB script to segment and track fusing droplets and calculate their minor and major axis over time. The ratio between the major and minor axis of the fusing droplets over time was fitted to a single exponential equation:

$$f\left( t \right) = A \times \exp \left( { - \frac{t}{\tau }} \right) + c$$

where c represents the offset to a perfect sphere. The inverse capillary velocity, the ratio between dynamic viscosity and surface tension, was estimated using a linear fit of the time constant (*τ*) for every fusion event against the geometric radius of the fusing droplets ($r=\sqrt{r_{1}\times r_{2}}$) using the following equation:

$$\tau \left( r \right) = a \times r$$

where *a* indicates the slope of the line and represents the inverse capillary velocity.

### Dual-colour coacervates

Two populations of coacervates were prepared as for the FRAP experiments, with one population using Cy5-S1 and another using FAM-S1. After preparation, coacervates were incubated for 10 min. Coacervates with differently labelled S1 RNA were then mixed and 10 µl of Pico-Surf was added to generate a water–oil emulsion. Images were acquired using a Plan-Apochromat 63× NA 1.4 oil DIC objective and a Zeiss LSM 880 Airy inverted confocal microscope with a 32-channel Airy Scan detector. FAM fluorophore was excited by using an argon Multiline laser at *λ* = 405 nm and the Cy5 fluorophore with a *λ* = 633 nm laser. FAM and Cy5 images were captured independently using an AiryScan detector in SuperResolution acquisition mode.

After acquisition, images were analysed using a custom-made MATLAB script to measure the Pearson correlation coefficient between the Cy5 and FAM images for individual droplets. The Pearson coefficient was calculated with a built-in function (corr) that uses the following equation:

$$\rho\left( {a,b} \right) = \frac{{\mathop {\sum }\nolimits_{i = 1}^n (X_{a,i} - \bar X_a)(Y_{b,i} - \bar Y_b)}}{{\left\{ {\mathop {\sum }\nolimits_{i = 1}^n (X_{a,i} - \bar X_a)^2\mathop {\sum }\nolimits_{j = 1}^n (Y_{b,j} - \bar Y_b)^2} \right\}^{1/2}}}$$

where *X* and *Y* represent two different image matrices, *a* and *b* represent a column in matrix *X* and *Y*, repectively.

### ICP-MS

Samples were prepared as for the turbidity assays and then centrifuged for 30 min at 20,000 r.c.f. After centrifugation, the coacervate phase was separated from the supernatant for acidification. Then, 500 μl of high-purity 1% (v/v) HNO_3_ was added to the coacervate phase and samples were sonicated for 15 min at room temperature. After sonication, samples were further diluted with 4.5 ml of 1% (v/v) HNO_3_ and analysed using a quadrupole ICP-MS NexION 350x (Perkin Elmer). The system was calibrated for magnesium with an ICP element standard solution VI (Merck). ICP curves were fitted to:

$$\begin{array}{l}f\left( x \right) = \sigma \times A_1 \times \exp \left( {\frac{{ - x - x_0}}{\tau }} \right) \times \exp \left( \left( {\frac{{\sigma ^2}}{{4 \times \tau ^2}}} \right)\right.\\\qquad\quad\left. \times \left( {1 + {{{\mathrm{erf}}}}\left( {\left( {\frac{{x - x_0}}{\sigma }} \right) - \left( {\frac{\sigma }{{2 \times \tau }}} \right)} \right)} \right) \right)\end{array}$$

### ITC

ITC was performed using a microcalorimeter (MicroCal PEAQ-ITC, Malvern Panalytical). The reference power of the cell was adjusted to 5 μcal s^−1^. ITC was first performed on Mg^2+^ binding to torula yeast RNA. For this purpose, torula yeast RNA in 10 mM Tris 7.5 was loaded at different concentrations in the measuring cell. Several torula yeast concentrations in nucleotide units were used: 10, 7.5, 5 and 2.5 mM. A solution containing 20 mM MgCl_2_ and 10 mM Tris 7.5 was titrated into the cell. The first injection introduced 0.2 μl and was discarded, as specified in the manufacturer’s instructions. The subsequent 39 injections had a volume of 1 μl with 200 s between injections. If the curve was not completed after 40 injections, the syringe was refilled and a new experiment was started without changing the material in the cell. This increases the final molar ratio attained. The curves of Mg^2+^ titration to pure buffer were subtracted from the curve derived from a Mg^2+^ binding to RNA. The total heat for the interaction was calculated as the sum of the heats released per injection and then normalized by the moles of RNA in the cell.

To measure heat release upon peptide binding in the presence or absence of Mg^2+^, the ITC cell was filled with torula yeast RNA in 10 mM Tris 7.5 with or without 5 mM MgCl_2_. We then titrated a solution of 10 mM R_9_, 10 mM K_9_ or 100 mM (RGG)_4_ in 10 mM Tris 7.5 with or without MgCl_2_ to the cell containing the torula yeast RNA. Torula yeast was present at different concentrations from 0.4 mM to 0.9 mM. The first injection introduced 0.2 μl and was discarded. The subsequent 39 injections had a volume of 1 μl with 200 s between injections for R_9_ and K_9_ and 300 s for (RGG)_4_. The heat released in the presence of MgCl_2_ was subtracted from the heat released in the absence of MgCl_2_ for every peptide. This difference was then compared to the heat released upon Mg^2+^ binding to RNA.

### Total droplet FRAP

(RGG)5 coacervates were prepared at a 40 a.a./nt ratio. The final mix contained 5 µM E, 6 µM S2, 0.1 µM Cy5-S1, 0.2 µM FITC-(RGG)_5_ and 17.5 mM (RGG)_5_ (in monomer units) in 10 mM Tris 7.5 and 20 mM MgCl_2_. A master mix containing all the RNA strands, MgCl_2_ and Tris was prepared before mixing the RNA with the peptide to ensure that all Cy5-S1 reacted to form Cy5-P (the product of the ligation). After mixing with the peptide solution, samples were incubated at room temperature for 1 min. After incubation, 5 μl of Pico-Surf was added to form a water–oil emulsion. The samples were imaged using a Plan-Apochromat 40×/1.2 water objective mounted on a Zeiss LSM 880 Airy inverted confocal microscope. Images were taken every 30 s for a total time of 63 min. Droplets were bleached using a 355 nm laser after the fifth frame. The Cy5 and FITC channels were imaged simultaneously.

For the analysis the droplets were segmented using the FITC–peptide channel, which recovered immediately after exposure to the 355 nm laser. The generated mask was then used to monitor Cy5 fluorescence in the droplets over time.

## Online content

Any methods, additional references, Nature Research reporting summaries, source data, extended data, supplementary information, acknowledgements, peer review information; details of author contributions and competing interests; and statements of data and code availability are available at 10.1038/s41557-022-00890-8.

## Supplementary information

Supplementary Information Supplementary Figs. 1–17, in–out diffusion of RNA from coacervates, references, complete gels.

## Data Availability

The data supporting the findings of this study are available within the paper and its Supplementary Information. Numeric data underlying all plots in the main publication and the extended data are available as source data files. Image data in the form of images (gels) is provided in the Supplementary Information. All movie and image raw data is archived on magnetic tape at the Max Planck Institute of Molecular Cell Biology and Genetics, Dresden and can be made available upon reasonable request. Source data are provided with this paper.

## Source data

Source Data Fig. 1 Annotated complete gels Fig. 1
Source Data Fig. 1 Statistical source data for Fig. 1
Source Data Fig. 2 Annotated complete gels Fig. 2f
Source Data Fig. 2 Statistical source data for Fig. 2
Source Data Fig. 3 Statistical source data for Fig. 3
Source Data Fig. 4 Statistical source data for Fig. 4
Source Data Fig. 5 Statistical source data for Fig. 5
Source Data Fig. 6 Statistical source data for Fig. 6
Source Data Extended Data Fig. 1 Statistical source data for Extended Data Fig. 1
Source Data Extended Data Fig. 2 Statistical source data for Extended Data Fig. 2
Source Data Extended Data Fig. 3 Statistical source data for Extended Data Fig. 3
Source Data Extended Data Fig. 4 Statistical source data for Extended Data Fig. 4

## Extended data

are available for this paper at 10.1038/s41557-022-00890-8.

## Code availability

The MATLAB codes used to analyse the data can be found at https://git.mpi-cbg.de/jiglesia/iglesias_et_al.
