# Supplementary material for: Charge-density reduction promotes ribozyme activity in RNA–peptide coacervates via RNA fluidization and magnesium partitioning
Source: Nat Chem. 2022 Feb 14;14(4):407–16. doi: 10.1038/s41557-022-00890-8 (PMC8979813; doi:10.1038/s41557-022-00890-8)

nature portfolio

https://doi.org/10.1038/s41557-022-00890-8

## Supplementary information

# Charge-density reduction promotes ribozyme activity in RNA–peptide coacervates via RNA fluidization and magnesium partitioning

In the format provided by the authors and unedited

# Supplementary Information

Charge density reduction promotes ribozyme activity in RNA-peptide protocells via fluidization and magnesium partitioning

Juan M. Iglesias-Artola, Björn Drobot, Mrityunjoy Kar, Anatol W. Fritsch, Hannes Mutschler, T-Y Dora Tang, Moritz Kreysing

**Materials** ..... **2**

General reagents ..... 2

Peptides ..... 2

Nucleic acids ..... 3

RNA sequences ..... 3

DNA sequences ..... 3

**Supplementary Figure** ..... **4**

Supplementary Fig. 1 | $Mg^{2+}$ is required for RNA catalytic activity. ..... 4

Supplementary Fig. 2 | $R_n$ coacervates are stable under peptide excess. ..... 5

Supplementary Fig. 3 | Differential dissolution of $K_n$ coacervates under peptide excess. ..... 6

Supplementary Fig. 4 | $(RGG)_n$ coacervates are stable under peptide excess. ..... 7

Supplementary Fig. 5 | $R_n$ peptides completely inhibit ribozyme activity from coacervation onset. ..... 8

Supplementary Fig. 6 | $K_n$ peptides show residual RNA enzymatic activity at low amino acid-to-nucleotide ratios. ..... 9

Supplementary Fig. 7 | $(RGG)_4$ shows reduced activity in the coacervate phase. ..... 10

Supplementary Fig. 8 | Cy5-P RNA shows reduced or no mobility inside $R_n$ coacervates. ..... 10

Supplementary Fig. 9 | Cy5-P RNA shows mobility inside $K_n$ coacervates. ..... 11

Supplementary Fig. 10 | Cy5-P diffusion coefficient is maximal in $(RGG)_4$ coacervates. ..... 11

Supplementary Fig. 11 | RNA-$(RGG)_5$ coacervates show liquid-like properties. ..... 12

Supplementary Fig. 12 | $K_n$ coacervates permit RNA-RNA interactions. ..... 13

Supplementary Fig. 13 | Charge interspacing shifts turbidity curve to higher aa/nt ratios. ..... 14

Supplementary Fig. 14 | $(RGG)_5$ coacervates recover enzymatic activity in the presence of 20 mM $MgCl_2$. ..... 15

Supplementary Fig. 15 | RNA in and out diffusion is limited in $(RGG)_5$ coacervates. ..... 16

Supplementary Fig. 16| RNA diffusion coefficient changes along the transition regime. ..... 17

Supplementary Fig. 17| $(KGG)_9$ coacervates can sustain ribozyme activity at 20 mM $MgCl_2$. ..... 18

**In-out-diffusion of RNA from coacervates** ..... **19**

**Supplementary References** ..... **22**

# Materials

## General reagents

Trizma base (121.14 g/mol), hydrochloric acid 1.0 N (36.46 g/mol) ethylenediaminetetraacetic acid disodium salt (EDTA, 372.24 g/mol), guanidinium thiocynate (118.16 g/mol), formamide (45.04 g/mol), tetramethylethylendiamine (TEMED, 116.21 g/mol), Orange G (452.37 g/mol), ammoniumperoxodisulfate (APS, 228.20 g/mol), sodium dodecyl sulfate (288.372 g/mol), spermidine (145.25 g/mol), dithiothreitol (DTT, 154.25 g/mol), glacial acetic acid (60.05 g/mol) were purchased from Sigma-Aldrich. Magnesium chloride hexahydrate (203.30 g/mol), sodium hydroxide (39.997 g/mol), boric acid (61.83 g/mol) and urea (60.06 g/mol) were purchased from Merck. Rotiphorese Gel 40 (40% solution mix of 19:1 Acrylamide-Bisacrylamide) was purchased from Carl Roth. Ribonucleoide solution mix (N0466) and inorganic pyrophosphatase from *E.* coli (M0361), were purchase from New England BiolLabs (NEB). Nuclease-free water, not DEPC-treated, was purchased from Ambion. 2% Pico-Surf™ 1 in Novec™ 7500 was purchased from Sphere Fluidics. All reagents were used without further purification.

## Peptides

All peptides with a defined length were purchased from Peptides&Elephants as chloride salts with at least 95% purity, including: $K_5$ (658.80 g/mol), $K_6$ (787.06 g/mol), $K_7$ (915.20 g/mol), $K_8$ (1043.40 g/mol), $K_9$ (1171.50 g/mol), $K_{10}$ (1299.77 g/mol), $K_{18}$ (2325.00 g/mol), $R_3$ (486.60 g/mol), $R_4$ (642.75 g/mol), $R_5$ (789.96 g/mol), $R_7$ (1111.3 g/mol), $R_9$ (1423.71 g/mol),$R_{10}$ (1579.90 g/mol), $R_{18}$ (2829.41 g/mol), $(RGG)_3$ (828.9 g/mol), $(RGG)_4$ (1099.18 g/mol), $(RGG)_5$ (1369.48 g/mol). Stock solutions where prepared by dissolving the peptides in nuclease-free water. To calculate the monomer molecular weight it was assumed that every charged amino acid (arginine or lysine) and the N-terminus of the peptide were neutralized by a chloride counterion. The average amino acid molecular weight was calculated as follows:

$$AA_{MW} = \frac{MW_{peptide}}{n_{AA}} + \frac{MW_{Cl^-} \times n_{Cl^-}}{n_{AA}}$$

where $AA_{MW}$ is the average amino acid molecular weight, $MW_{peptide}$ the peptide molecular weight, $MW_{Cl}$ the chloride molecular weight (35.453), $n_{AA}$ the number of amino acids, and $n_{Cl}$ the number of chloride counterions. With the averaged monomer molecular weight, the volume required in µL for resuspension was calculated using the following equation:

$$V = \frac{m_{peptide}}{FC \times AA_{MW}} \times 1000$$

where $FC$ is the final concentration in mM and $m_{peptide}$ the mass in mg of peptide. Poly-R (5 – 15 kDa, monomer: 191.65 g/mol) and Poly-K (4– 15 kDa, monomer: 209.09 g/mol) were purchased from Sigma Aldrich as hydrochloride or hydrobromide salts, respectively. The pH of all peptide solutions was adjusted to between 7 and 7.5 using NaOH and Merck pH-indicator solution (109175) to verify the pH.

## Nucleic acids

RNA sequences for the R3C ligase system were obtained from Lincoln and Joyce (2009)[1]. S1 and S2 RNAs correspond to A' and B'. Fluorescently labelled RNAs were purchased from Biomers or Integrated DNA technologies (IDT); these include FAM-E, FAM-S1, FAM-S2, Cy5-$(AC)_9$, Cy5-S1, Cy5-S2. Lyophilized RNA was resuspended in RNA-free water (Ambion) to a stock concentration of 100 µM. Torula yeast RNA type VI was purchased from Sigma-Aldrich. DNAs used for *in vitro* transcription were purchased from Biomers.

## RNA sequences

| Name | Sequence |
|---|---|
| FAM-E | 5′ 6-FAM-GGUUCAUGUGCUCGAUUGUUACGUAAGU AACAGUUUGAAUGGGUUGAAGAAUGAGACCGCAACUU A 3′ |
| FAM-S1 | 5′ 6-FAM-GGAAGUUGUGCUCGAUUGUUCUAAGGUA ACUUAGAACAGUUUGAAUGGGUUGAAGUUU 3′ |
| FAM-S2 | 5′ 6-FAM-GAGACCGCAUGAAUAUUC 3′ |
| Cy5-$(AC)_9$ | 5′ Cy5-ACACACACACACACACAC 3′ |
| Cy5-S1 | 5′ Cy5-GGAAGUUGUGCUCGAUUGUUCUAAGGUAAC UUAGAACAGUUUGAAUGGGUUGAAGUUU 3′ |
| Cy5-S2 | 5′ Cy5-GAGACCGCAUGAAUAUUC 3′ |

## DNA sequences

| Name | Sequence |
|---|---|
| E | 5′ TAAGTTGCGGTCTCATTCTTCAACCCATTCAAAT GTTACTTACGTAACAATCGAGCACATGAACCTATAGT GAGTCGTATTAATTTC 3′ |
| S2 | 5′ GAATATTCATGCGGTCTCTATAGTGAGTCGTATT AATTTC 3′ |
| T7-Prom | 5′ GAAATTAATACGACTCACTATAG 3′ |

## Supplementary Figure

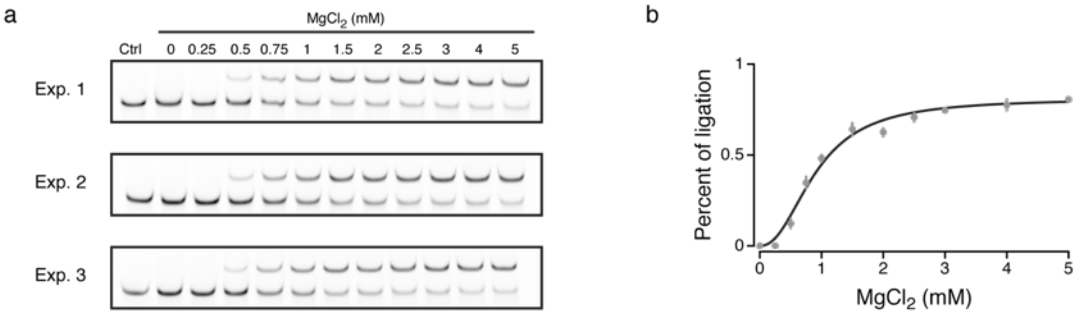


**Supplementary Fig. 1 | $Mg^{2+}$ is required for RNA catalytic activity.**

**a,** 12.5% polyacrylamide gels of $MgCl_2$ titration to 5 µM E, 6 µM S2 and 0.1 µM Cy5-S1. Three independent repeats were carried out and reactions were incubated for 1 hour. Control lanes show the starting substrate. **b,** Quantification of gels on (**a**); data are shown as the mean ± 95% CI. Data points were fitted to a Hill equation.

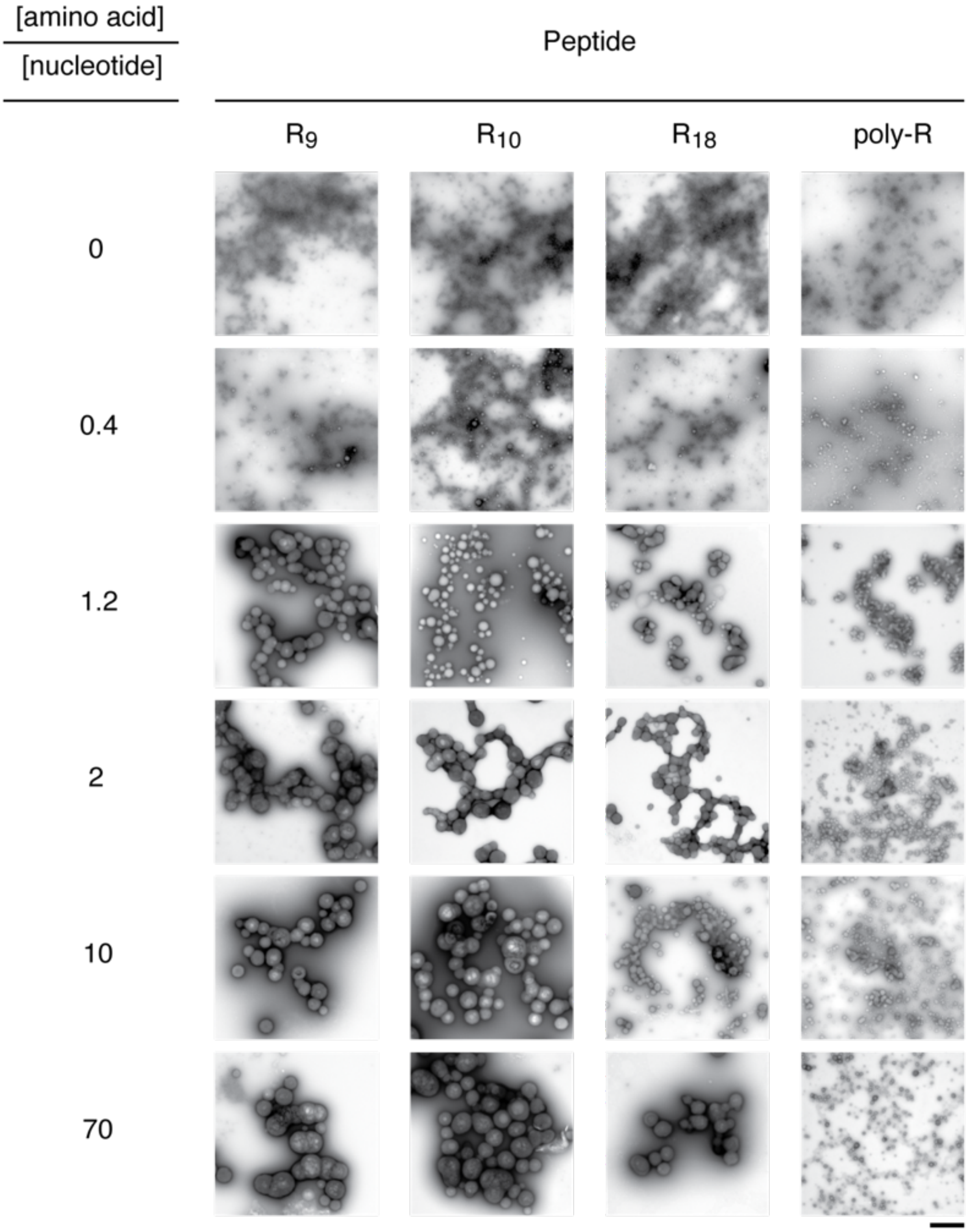


**Supplementary Fig. 2 | $R_n$ coacervates are stable under peptide excess.**

Transmission electron micrographs of $R_9$, $R_{10}$, $R_{18}$ and poly-R show that coacervates are still present at high peptide excess. Scale bar: 1 µm.

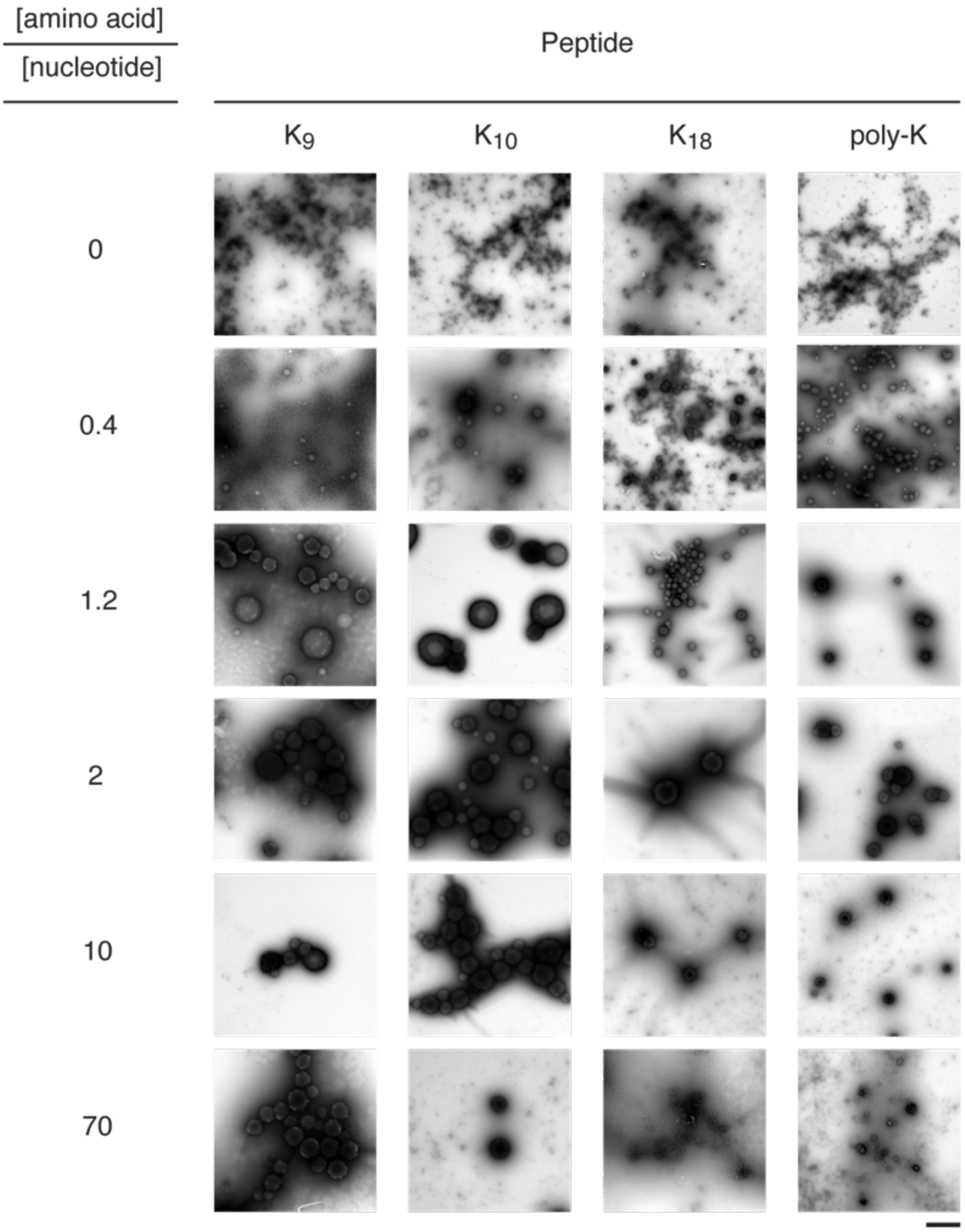


**Supplementary Fig. 3 | Differential dissolution of $K_n$ coacervates under peptide excess.**

Transmission electron micrographs show that $K_9$, $K_{10}$, $K_{18}$ and poly-K. $K_9$ coacervates are stable and do not dissolve under peptide excess. Peptides longer than $K_9$ result in coacervates that dissolve at high peptide ratios. Dissolution is accompanied by the formation of small filamentous structures, indicating the formation of soluble RNA-peptide complexes. Scale bar: 1 µm.

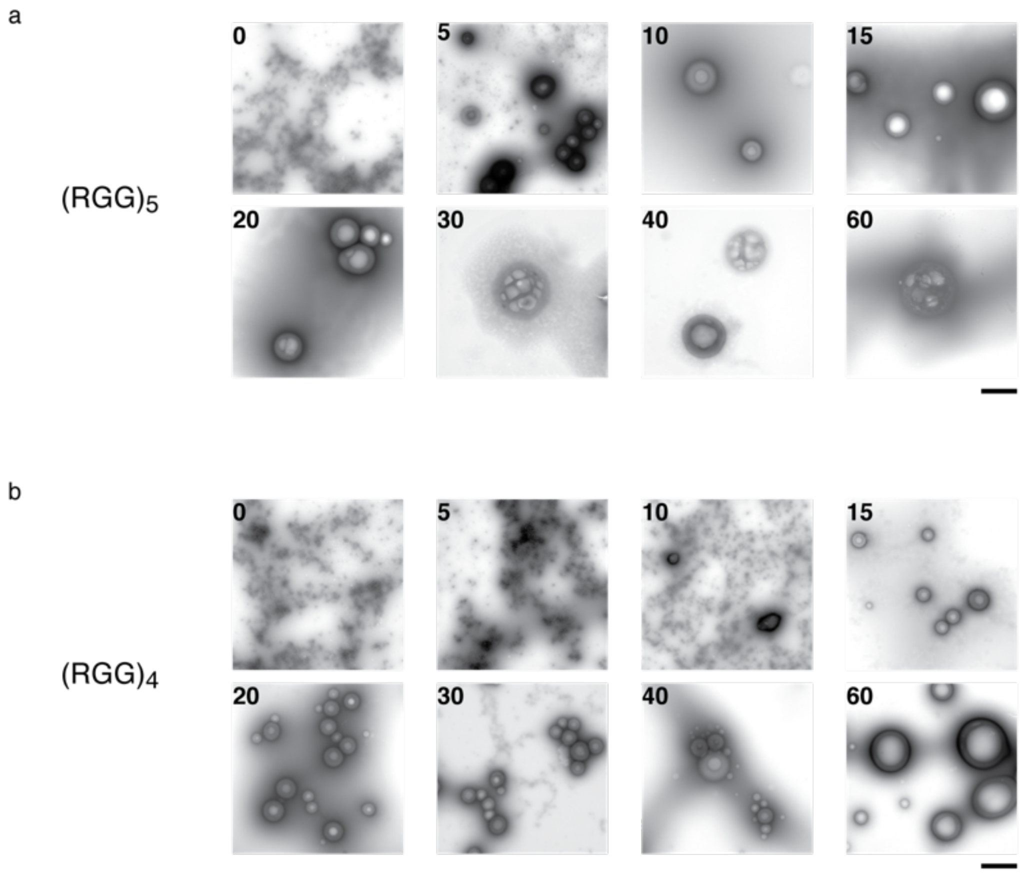


**Supplementary Fig. 4 | $(RGG)_n$ coacervates are stable under peptide excess.**

Transmission electron micrographs of (**a**) $(RGG)_5$ and (**b**) $(RGG)_4$. For $(RGG)_4$ and $(RGG)_5$, greater than 1:1 charge ratios are needed to trigger phase separation. Numbers in the top left corner of the micrographs represent the amino acid-to-nucleotide ratio used for sample preparation. Scale bar: 1 µm.

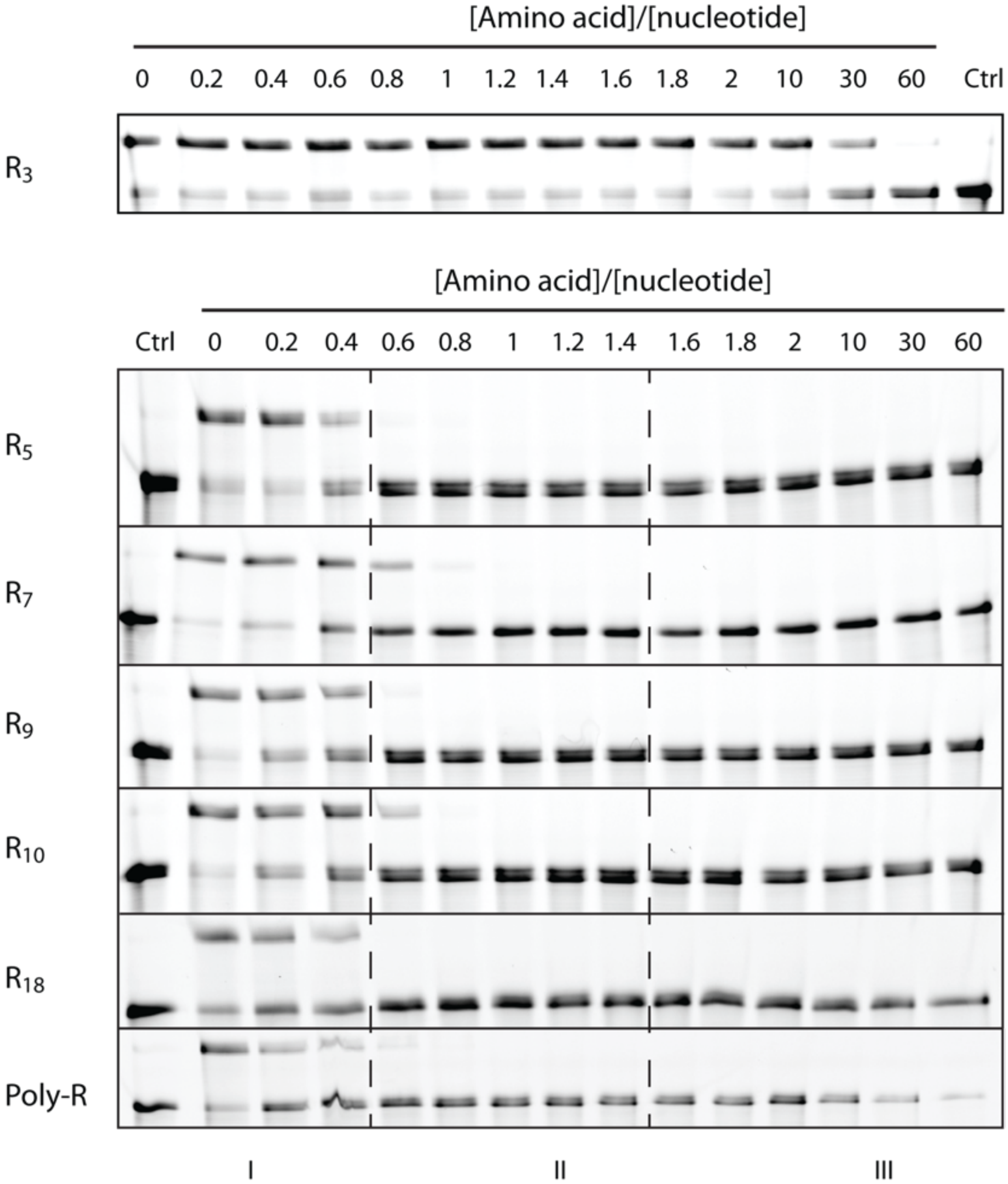


**Supplementary Fig. 5 | $R_n$ peptides completely inhibit ribozyme activity from coacervation onset.**

12.5% polyacrylamide 8M urea gels of R3C ligation at different amino acid-to-nucleotide ratios. $R_3$ (top panel) does not form coacervates and the reaction is not inhibited until high peptide excess. Gels for peptides that make coacervates (bottom panel) have been divided into three regions: (I) before turbidity onset, (II) region where turbidity increases, (III) stable turbidity regime. Ctrl lane shows starting material before incubation. Control lanes (Ctrl) show the starting substrate at time 0.

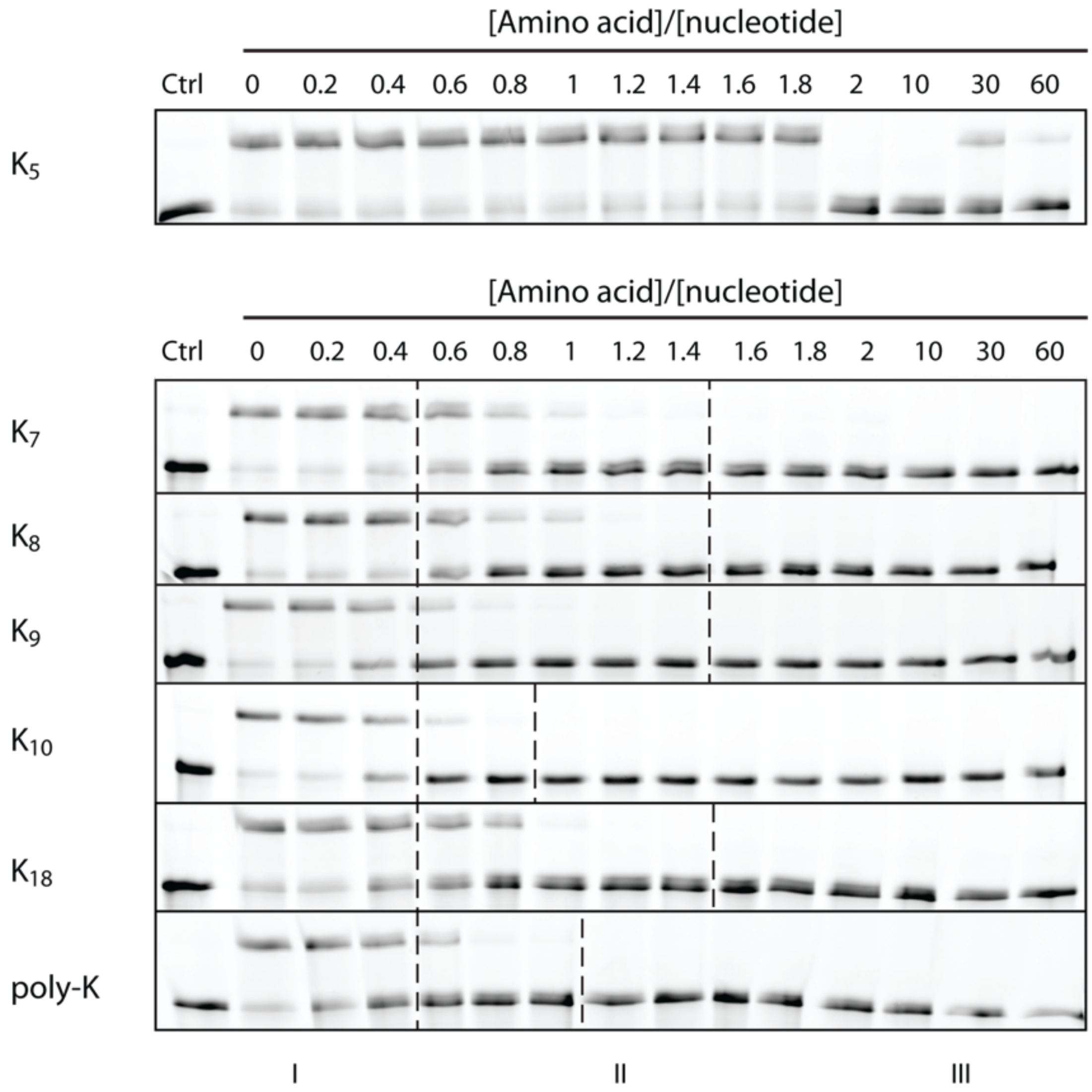


**Supplementary Fig. 6 | $K_n$ peptides show residual RNA enzymatic activity at low amino acid-to-nucleotide ratios.**

12.5% polyacrylamide 8M urea gels of R3C ligation at different amino acid-to-nucleotide ratios. $R_3$ (top panel) does not form coacervates and the reaction is not inhibited until high peptide excess. Gels for peptides that make coacervates (bottom panel) have been divided into three regions: (I) before turbidity onset, (II) region where turbidity increases, (III) stable turbidity regime. Ctrl lane shows starting material before incubation. Control lanes (Ctrl) show the starting substrate at time 0.

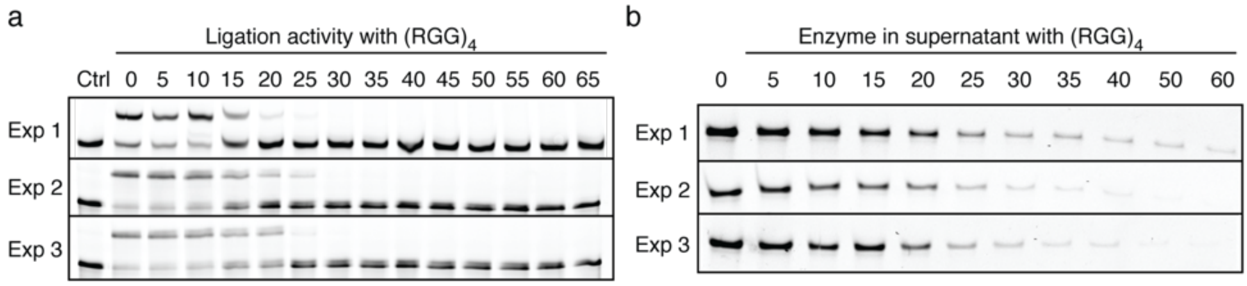


**Supplementary Fig. 7 | $(RGG)_4$ shows reduced activity in the coacervate phase.**

**a,** Three experimental repeats of R3C ligation at different amino acid-to-nucleotide ratios of $(RGG)_4$. Control lane shows the starting substrate. **b,** Enzyme present in the supernatant after centrifugation of the samples prepared at different amino acid-to-nucleotide ratios of $(RGG)_4$.

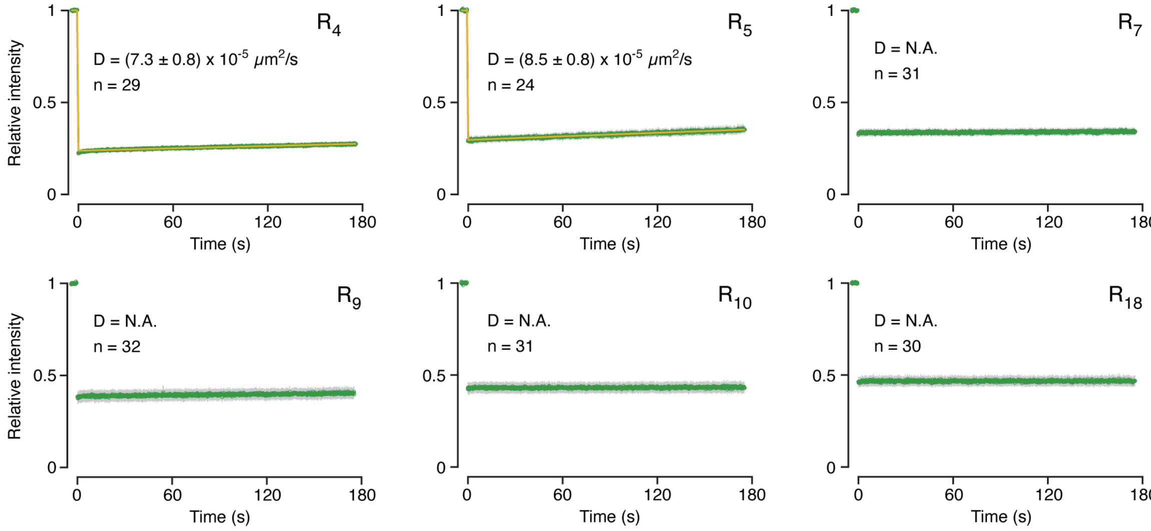


**Supplementary Fig. 8 | Cy5-P RNA shows reduced or no mobility inside $R_n$ coacervates.**

FRAP curves for $R_n$ coacervates show the mean ± 95% CI of n experiments with the 95% confidence interval as a shaded region. In $R_4$ and $R_5$ coacervate RNA diffusion was fitted to a single exponential curve assuming total RNA recovery. All other coacervates did not show RNA recovery after photobleaching. Apparent diffusion coefficients were derived from individual traces.

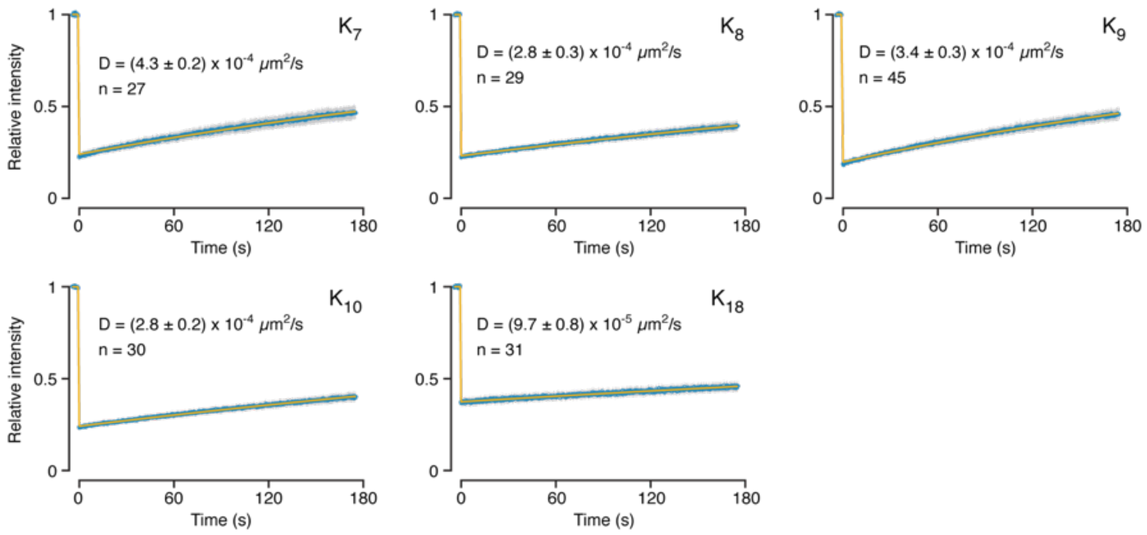


**Supplementary Fig. 9 | Cy5-P RNA shows mobility inside $K_n$ coacervates.**

FRAP curves for $K_n$ coacervates show the mean ± 95% CI of n experiments with the confidence interval as a shaded region. All curves were fitted to a single exponential assuming total RNA recovery. Apparent diffusion coefficients were derived from individual traces.

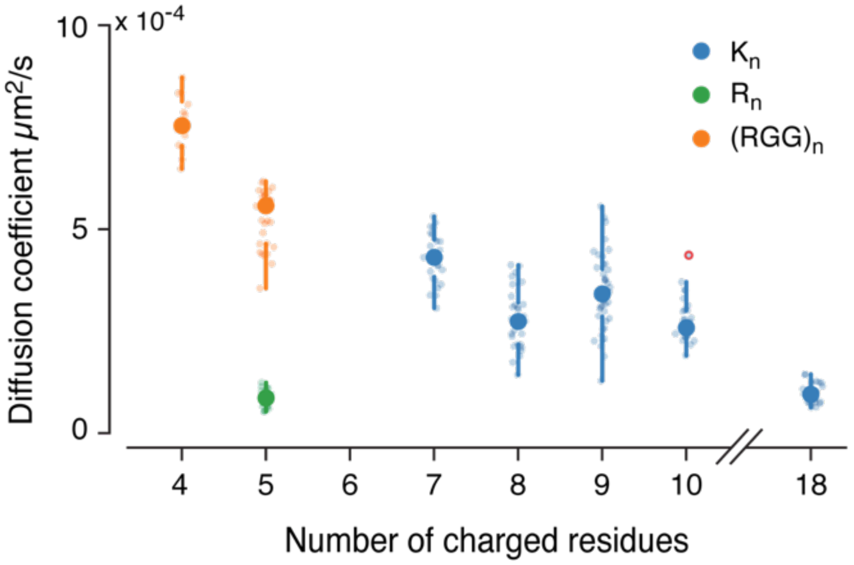


**Supplementary Fig. 10 | Cy5-P diffusion coefficient is maximal in $(RGG)_4$ coacervates.**

Boxplot of Cy5-P diffusion coefficients with filled circle representing the median, the space between the circles and the lines the 2nd and 3rd quartile, and the lines the 1st and 4th quartile. Scatter points represent independent observations. n is specified for each coacervate in Fig. 3 and Supplementary Fig. 8-10.

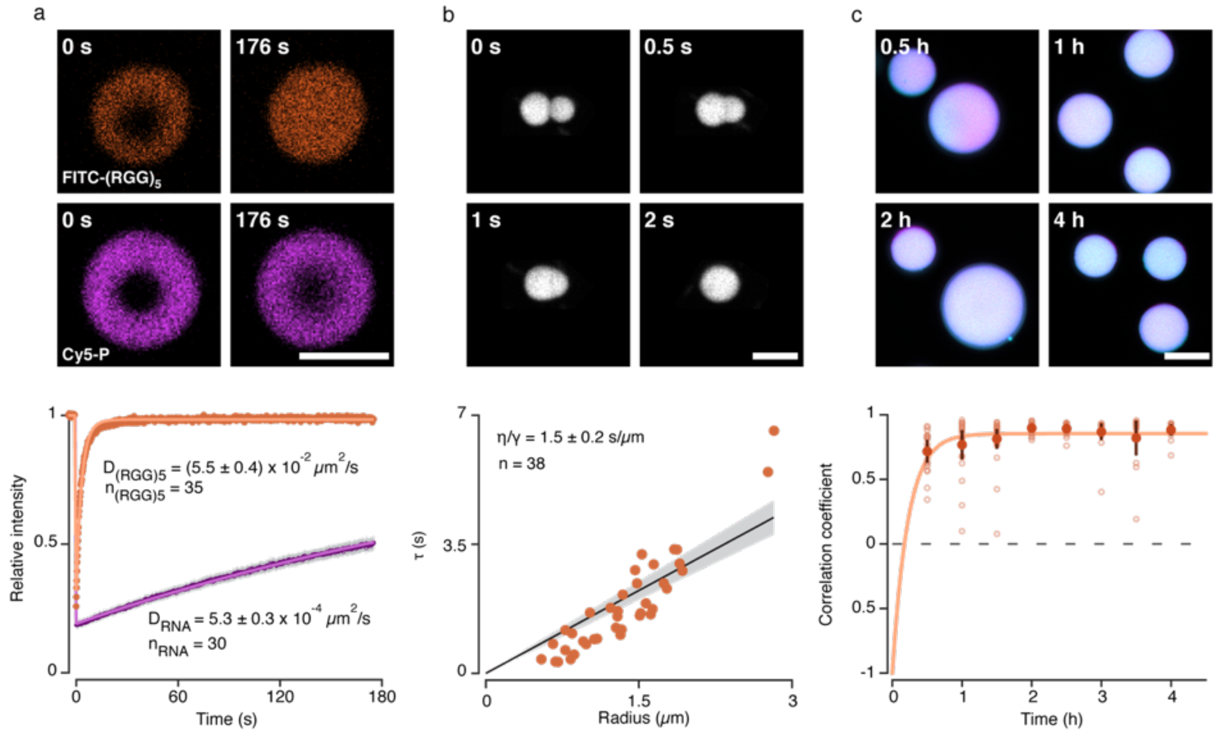


**Supplementary Fig. 11 | RNA-$(RGG)_5$ coacervates show liquid-like properties.**

**a,** FRAP experiments of FITC-$(RGG)_5$ and Cy5-P show differential mobility of the RNA and peptide components of the coacervate phase and increased RNA mobility compared to $K_n$ peptides. Apparent diffusion coefficients were derived from individual traces. Data are shown as the mean of n individual determinations ± 95% CI. **b,** Droplet fusion experiments show liquid-like behaviour of the coacervate protocells. Inverse capillary velocity is reported as the mean ± 95% CI. **c,** Droplets prepared with Cy5-P or FITC-P show complete RNA rearrangement after fusion with droplets containing differently labelled RNA. Rearrangement is completed after 1 hour of incubation. Pearson correlation coefficients fitted to guide with a single exponential equation.

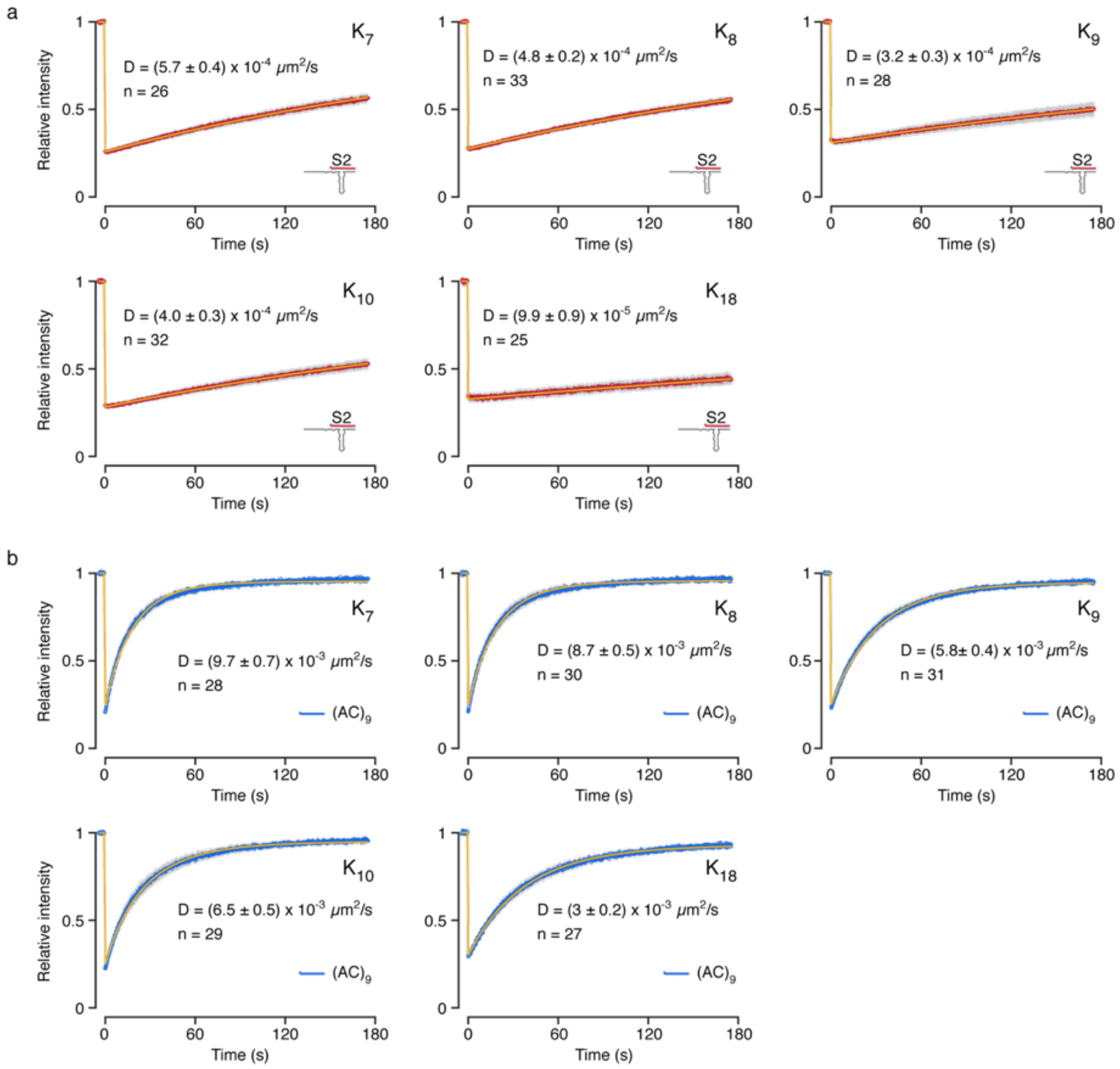


**Supplementary Fig. 12 | $K_n$ coacervates permit RNA-RNA interactions.**

**a,** FRAP curves of Cy5-S2 inside coacervates formed from $K_n$ peptides. **b,** FRAP curves of Cy5-$(AC)_9$, an RNA with the same nucleotide number as S2 but with no sequence complementarity with E, inside coacervates formed from $K_n$ peptides. Apparent diffusion coefficients were derived from individual traces. Cy5-S2 curves were forced to reach complete recovery; $(AC)_9$ has a free parameter for maximal recovery. Data are shown as the mean of n individual determinations; shaded areas represent 95% CI intervals.

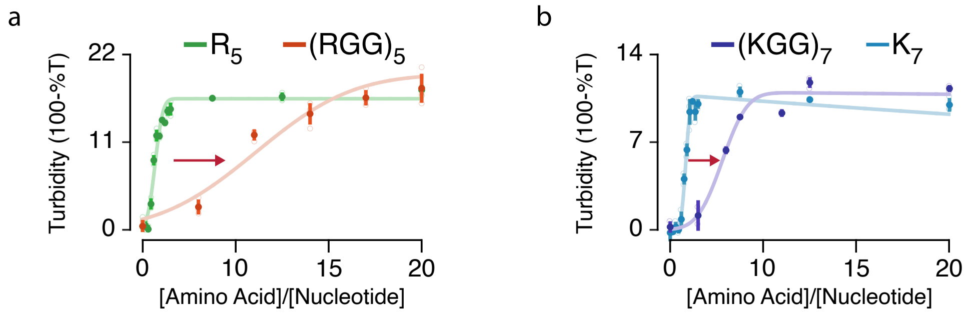


**Supplementary Fig. 13 | Charge interspacing shifts turbidity curve to higher aa/nt ratios.**

**a** Turbidity curves of $R_5$ (in green) and $(RGG)_5$ (in orange), against amino acid-to-nucleotide ratio. Samples contained 5 µM E and 6 µM S2 in 10 mM Tris pH 7.5 and 5 mM $MgCl_2$. **b** Turbidity curves of $K_7$ (in light blue) and $(KGG)_7$ (in purple), samples were prepared as for (**a**). n = 3 for all the curves; data are shown as the mean ± 68% confidence interval, open circles indicate individual observations.

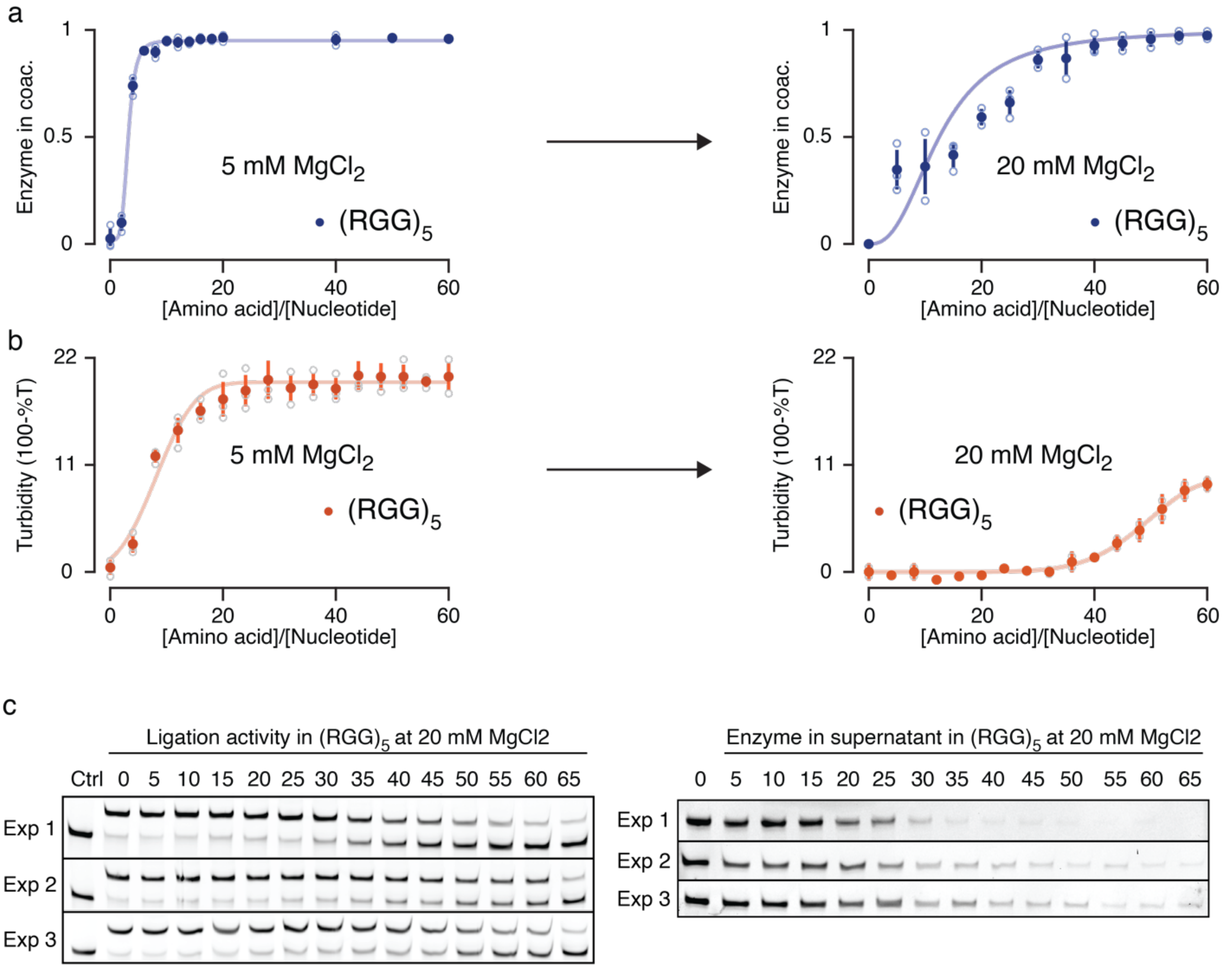


**Supplementary Fig. 14 | $(RGG)_5$ coacervates recover enzymatic activity in the presence of 20 mM $MgCl_2$.**

**a,** Sequestration data shows the ribozyme fraction within the coacervate phase, irrespectively of their size distribution at 5 mM $MgCl_2$ (left, n = 3) and 20 mM $MgCl_2$ (right, n = 3). Enzyme partition was measured after centrifugation. **b,** Turbidity plot of $(RGG)_5$ at 5 mM $MgCl_2$ (left, n = 3) and 20 mM $MgCl_2$ (right, n = 2). Control lane shows the starting substrate. Data are shown as the mean ± 68% CI, open circles indicate individual experiments. **c,** Three experimental repeats of R3C ligation at 20 mM $MgCl_2$ in the presence of $(RGG)_5$ (left panel) and enzyme present in the supernatant (right panel) at different aa/nt.

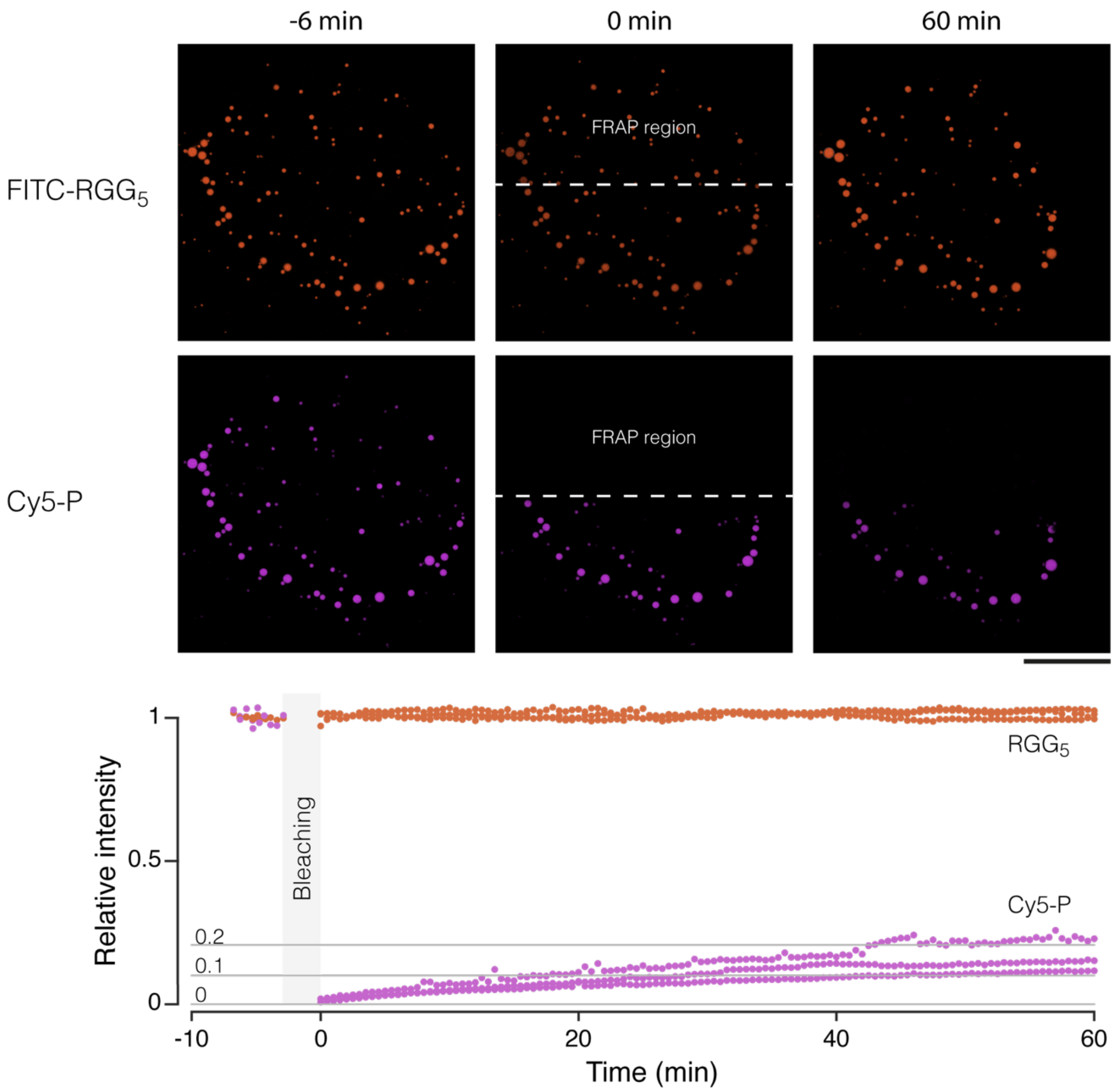


**Supplementary Fig. 15 | RNA in and out diffusion is limited in $(RGG)_5$ coacervates.**

Coacervates were prepared at a 40 times aa/nt ratio using 5 µM E, 6 µM S2, 0.1 µM Cy5-S1 and 0.1 µM FITC-$(RGG)_5$ in 10 mM Tris pH 7.5 and 20 mM $MgCl_2$. Coacervates were encapsulated using Pico-Surf. A emulsion droplet was chosen for the FRAP measurement and monitored for 60 min after bleaching. FITC and Cy5 images were acquired simultaneously. n = 3. Scale bar: 50 µm.

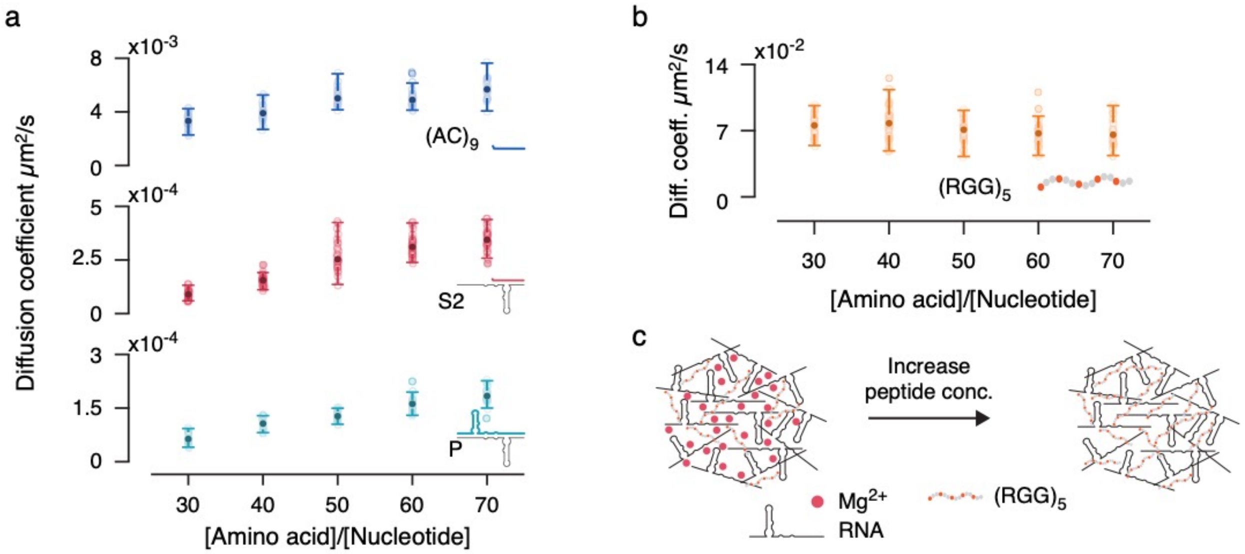


**Supplementary Fig. 16| RNA diffusion coefficient changes along the transition regime.**

**a,** Boxplot of diffusion coefficients for $(AC)_9$, S2 and P against amino acid-to-nucleotide ratio in $(RGG)_5$ coacervates prepared at 20 mM $MgCl_2$. The dot indicates the median, the lines the first and fourth quartile, and the spaces between the lines and the dot the second and third quartile. Individual observations are shown below the boxplot. Outliers are represented with dots surrounded by a thick line. Boxplots show that diffusion increases with increasing peptide concentration. **b,** Boxplot of the diffusion coefficients of the $(RGG)_5$ peptide against amino acid-to-nucleotide ratio. Peptide diffusion is independent of peptide concentration. **c,** Schematic of interactions inside the coacervate phase. At low amino acid-to-nucleotide ratios, $Mg^{2+}$ is able to interact with the RNA and crosslink it. At higher ratios, the peptide displaces $Mg^{2+}$ and reduces the number of RNA-RNA contacts**.**

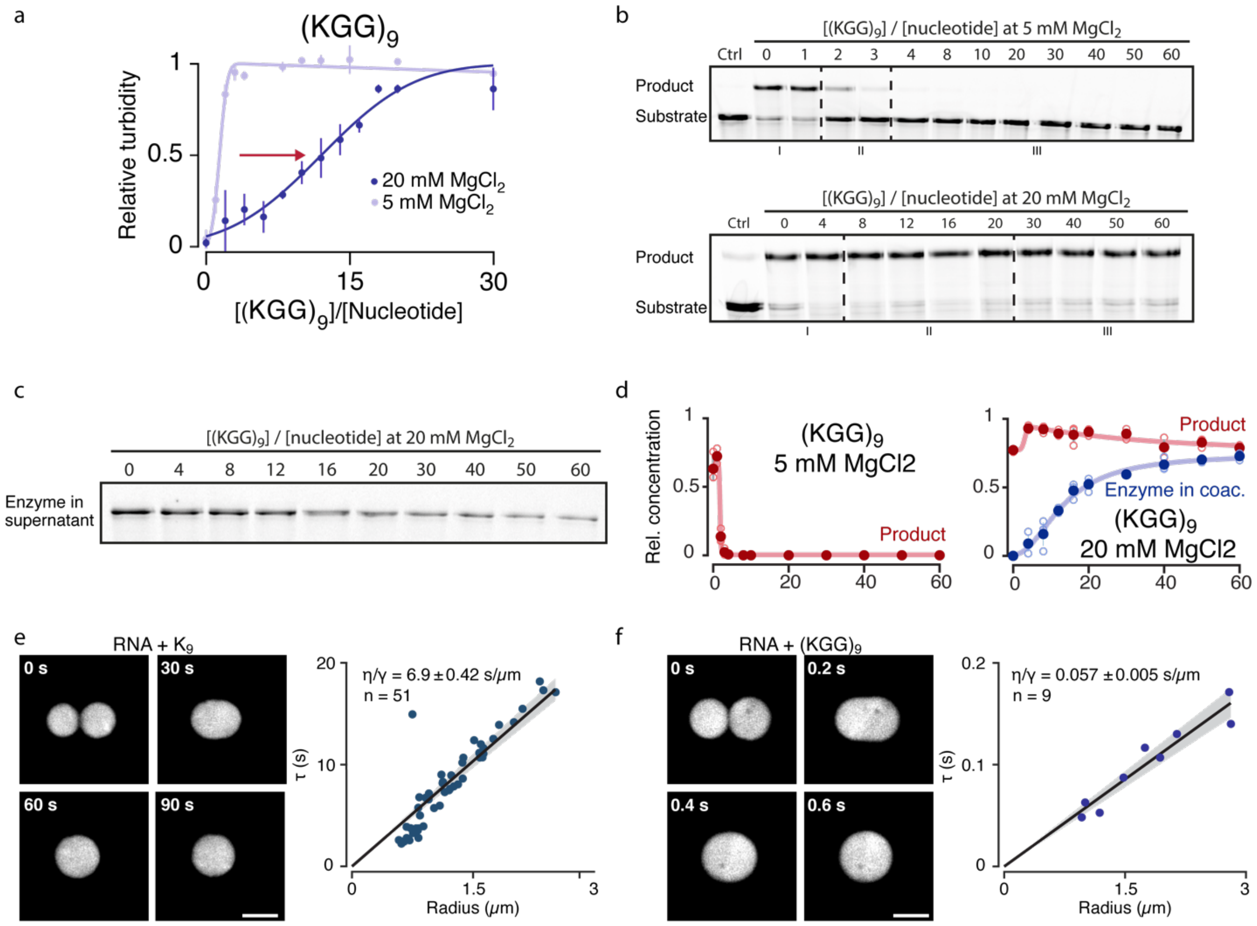


**Supplementary Fig. 17| (KGG)$_9$ coacervates can sustain ribozyme activity at 20 mM $MgCl_2$.**

**a,** Relative turbidity plots of (KGG)$_9$ prepared at 5 mM (light purple) and 20 mM (dark purple) mM $MgCl_2$. A right shift in the turbidity curve is observed when increasing $MgCl_2$ concentration, indicative of RNA-peptide interaction shielding. n = 3, error bars show the 95% confidence interval. **b,** R3C ligation activity in the presence of different aa/nt ratios of (KGG)$_9$ at 5 (top panel) and 20 (bottom panel) mM $MgCl_2$. (KGG)$_9$ coacervates can maintain enzymatic activity even at high peptide ratios. **c,** enzyme present in the supernatant at different aa/nt of (KGG)$_9$ in the presence of 20 mM $MgCl_2$. **d,** quantification of relative product formation (red lines) and relative enzyme concentration in the coacervate phase for (KGG)$_9$ coacervates prepared at 5 mM (left panel) and 20 mM (right panel) $MgCl_2$. n = 3 , error bars show the 68% confidence interval. **e-f,** Droplet fusion experiments further indicate a 120-fold difference in droplet fusion times between coacervates prepared with $K_9$ and those prepared with (KGG)$_9$. The plots show the characteristic relaxation time of fusion for two droplets plotted against their geometric length. Data were fitted with a linear fit while constraining the y intercept to 0. The inverse capillary velocity is inferred from the slope of the fit. The shaded region represents the 95% confidence interval of the fit. Scale bar: 5 µm

## In-out-diffusion of RNA from coacervates

To further substantiate the formation of production in coacervates, partition behaviour, remaining concentrations in solution as well as rate of in-out diffusion was considered in quantitative assays shown below.

1. From Supplementary Fig. 17b left panel we know that even at high $(RGG)_5$ concentrations the enzyme is active if coacervates are prepared at 20 mM $MgCl_2$.

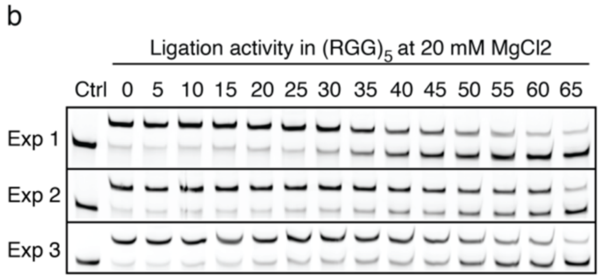


2. If we take 40 times (RGG)5 as an example. We see that under these conditions (20 mM MgCl2) between 0.018 (Exp 1) to 0.1 (Exp 3) of the enzyme is in the supernatant. This means that at least 90% of the enzyme is in the coacervate phase. Supplementary Fig. 17b right panel.

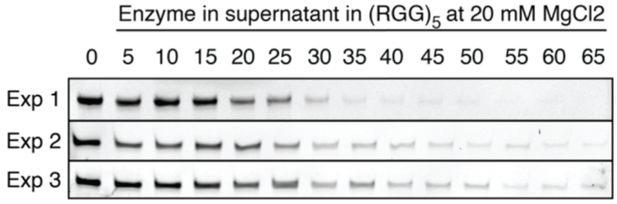


3. At this peptide concentration we obtain between 47% (Exp 1) to 72% (Exp 3) percent of ligated substrate after on 1h of incubation. Since we are using 100 nM of initial substrate this will translate to between 47 to 72 nM of product formed in the 5 µL reaction. Supplementary Fig. 17b left panel.

4. Since we know partitioning of the enzyme (66 nt long), we can estimate a partitioning for the S1 substrate (52 nt long), assuming they partition very similarly. This means that at least 90% of the substrate is in the coacervate phase. This translates to a concentration of not more than 10 nM in the supernatant (since we started with 100 nM).

5. If the reaction proceeds to 100% in the supernatant, we could get at most 10 nM of product formed. However, we observe 4 to 7 times this amount. This suggests that the reaction should happen in the coacervate phase, unless there is a rapid in-and-out exchange of RNA between the coacervate phase and the supernatant.

6. Such in out exchange can be observed for a fluorescently labelled $(RGG)_5$ peptide (see below). Upon photobleaching half of the field of view of the microscope, the $(RGG)_5$ distribution immediately recovers. This data additionally shows that small polymers quickly diffuse between regions to facilitate the signal recovery.

7. FRAP results on total droplets formed at 40 times (RGG)5 and 20 mM MgCl2 show that the exchange is not high enough to completely replenish the supernatant pool between 4 to 7 times. Supplementary Fig. 19).

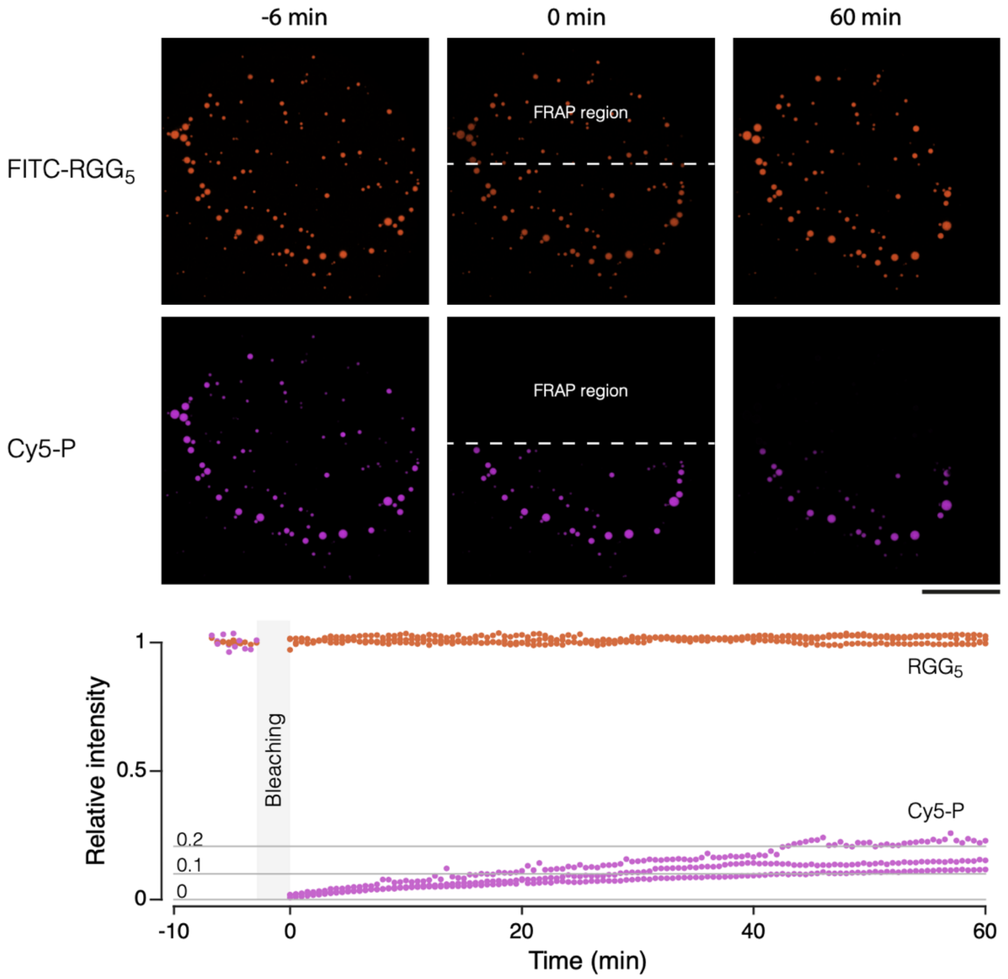

8. If we follow this argument for the rest of peptide concentrations we observe that even at the highest aa/nt ratio the amount of product formed highly exceeds what would be expected if only the supernatant fraction reacted **(a)**. In particular at high peptide excess (e.g. 60 fold) the product formation is about 50 times as high as can be expected by in/out diffusion and reaction in the buffer **(b)**.

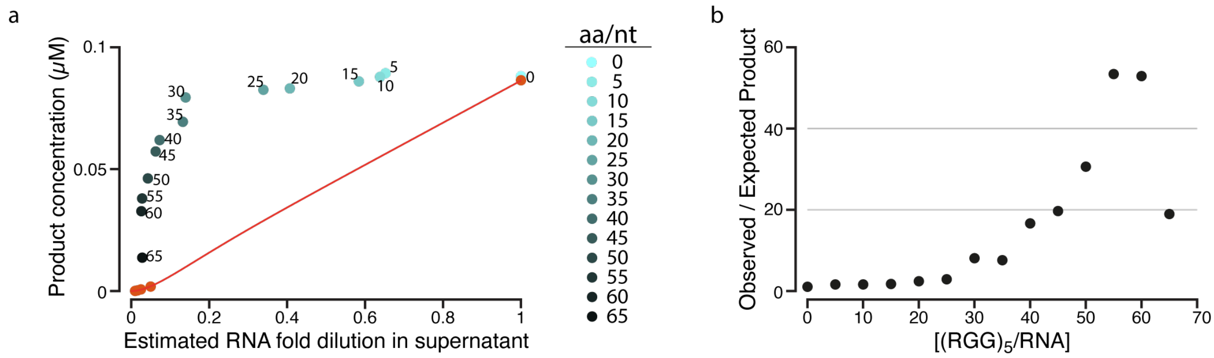


**RNA ligation occurs inside $(RGG)_5$ coacervates at 20 mM $MgCl_2$. a,** Product formation at different aa/nt ratios of $(RGG)_5$ exceeds the expected concentration if product was being formed in solution. The red line shows the concentration of product formed if the reaction proceeded at different RNA dilutions. The reaction dilutions were obtained from the amount of RNA-E remaining in the supernatant after coacervate centrifugation. A 1X dilution corresponds to 5 µM E, 0.1 µM S1 and 6 µM S2. The aa/nt ratios are shown as numbers next to the datapoints. n = 3. **b.** Ratio of observed over expected product if the R3C ligase reaction would take place in the supernatant.

## Supplementary References

1 Lincoln, T. A. & Joyce, G. F. Self-Sustained Replication of an RNA Enzyme. *Science* **323**, 1229 (2009).

Supplementary Fig. 1

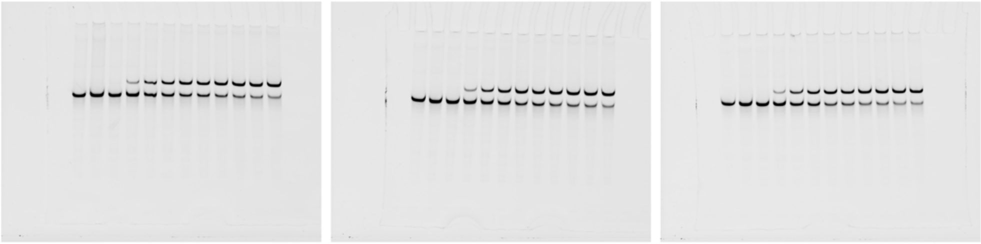

Supplementary Fig. 5

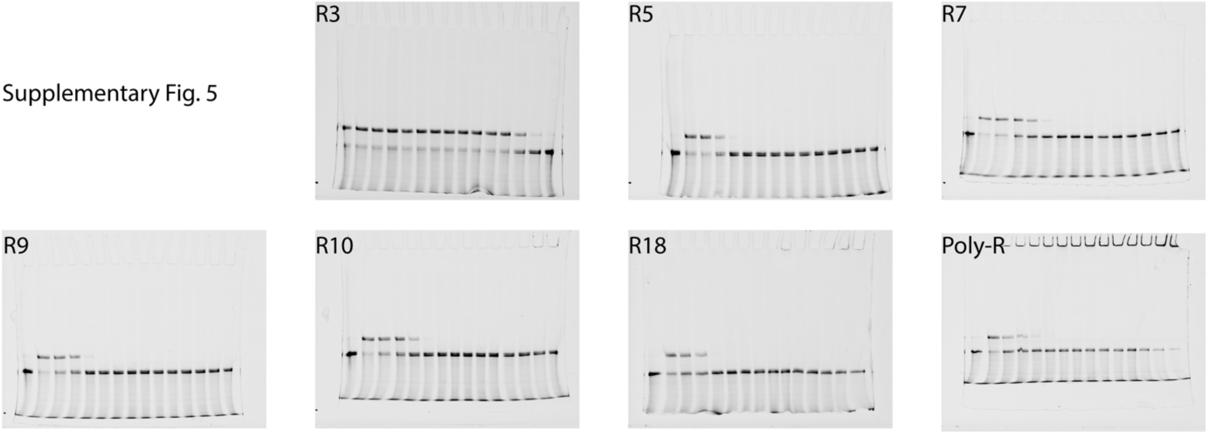


Supplementary Fig. 6

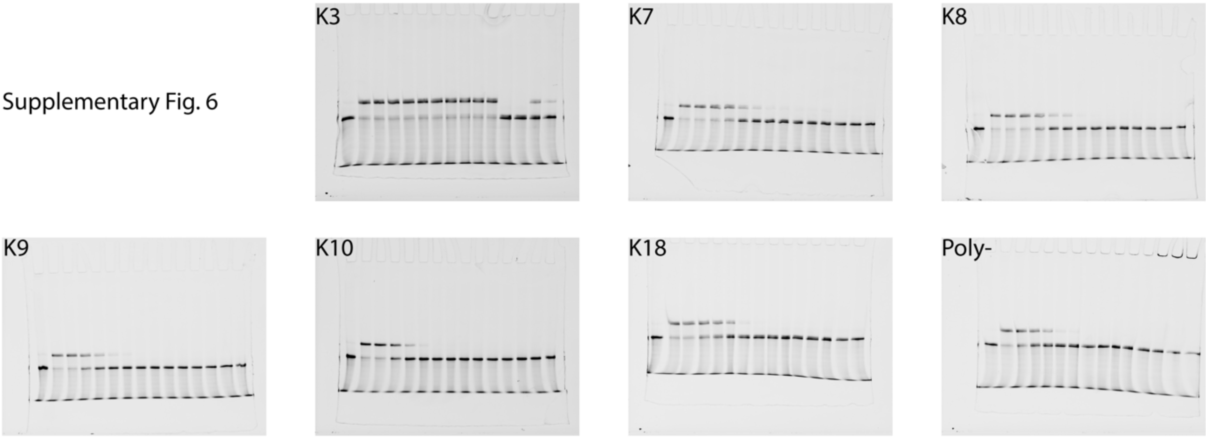

## Supplementary Fig. 7

Ligation activity
(RGG)4

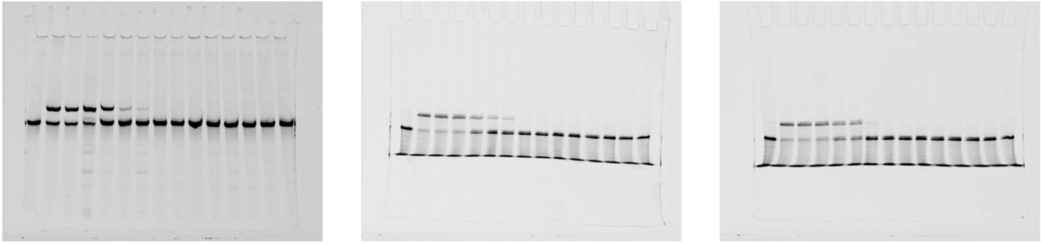

Enzyme in
supernatant
(RGG)4

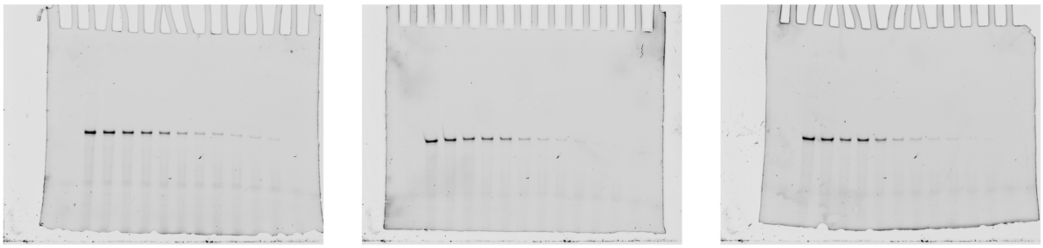

## Supplementary Fig. 14

Ligation activity
(RGG)5

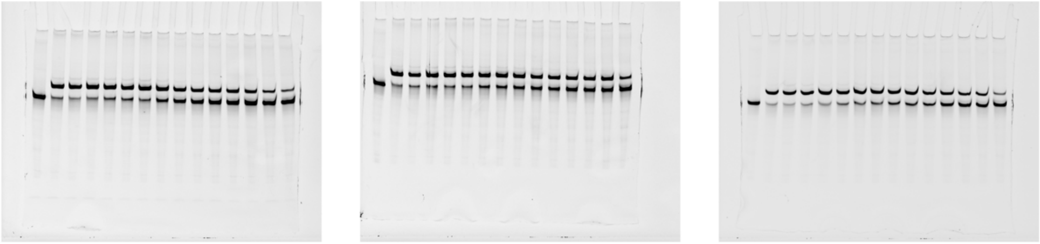

Enzyme in
supernatant
(RGG)5

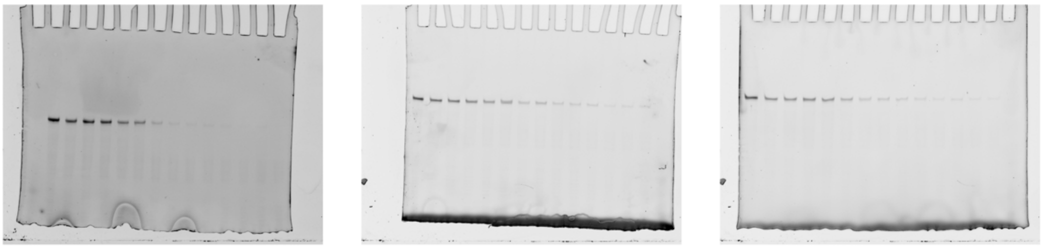

## Supplementary Fig. 17

Ligation activity (KGG)9, 5 mM $MgCl_2$

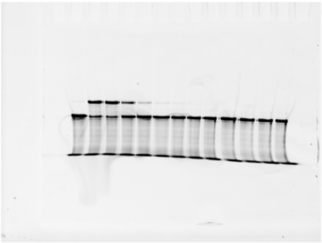

Ligation activity (KGG)9, 20 mM $MgCl_2$

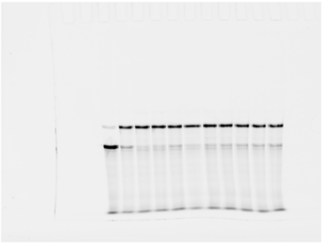

Enzyme in supernantant (KGG)9, 20 mM $MgCl_2$

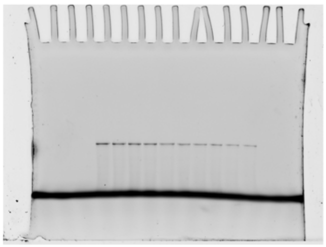

Supplement: Supplementary file 1 — Supplementary Figs. 1–17, in–out diffusion of RNA from coacervates, references, complete gels. [file 41557_2022_890_MOESM1_ESM.pdf]
