# Supplementary figures and images for: Charge-density reduction promotes ribozyme activity in RNA–peptide coacervates via RNA fluidization and magnesium partitioning
Source: Nat Chem. 2022 Feb 14;14(4):407–16. doi: 10.1038/s41557-022-00890-8 (PMC8979813; doi:10.1038/s41557-022-00890-8)

Fig 1d

R<sub>9</sub> Peptide

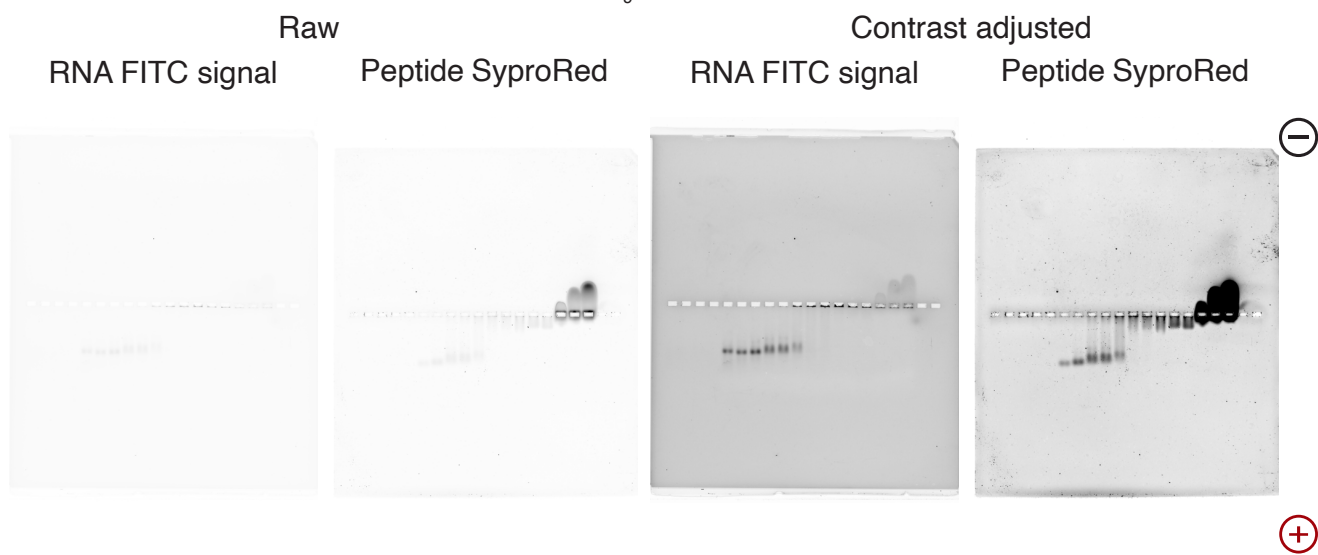

K<sub>9</sub> Peptide

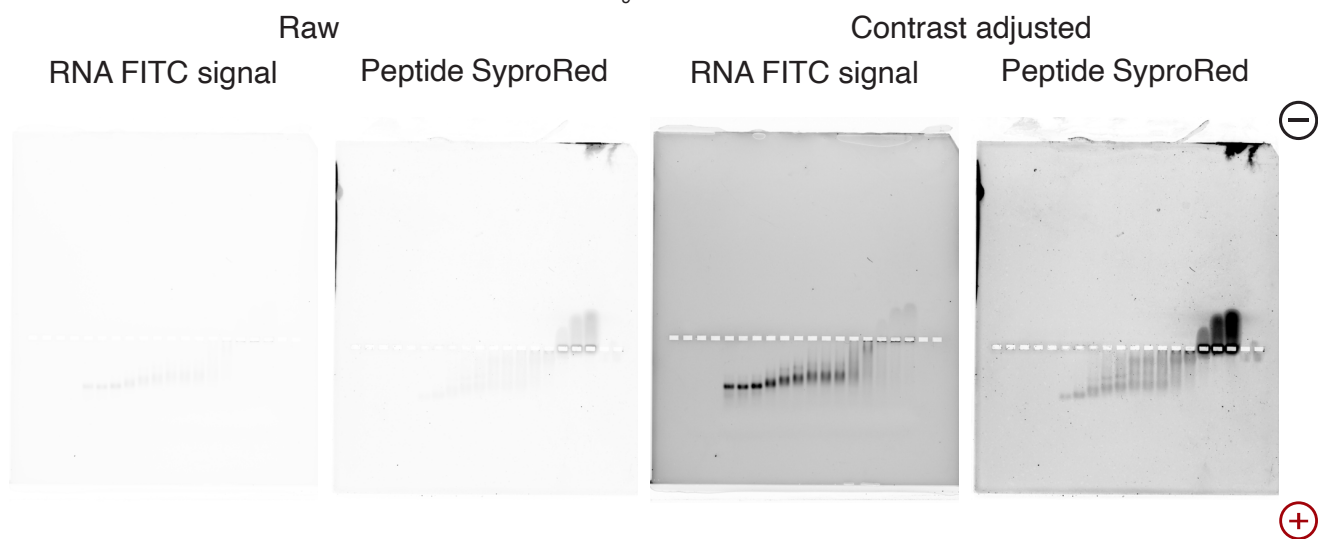

(RGG)<sub>4</sub> Peptide

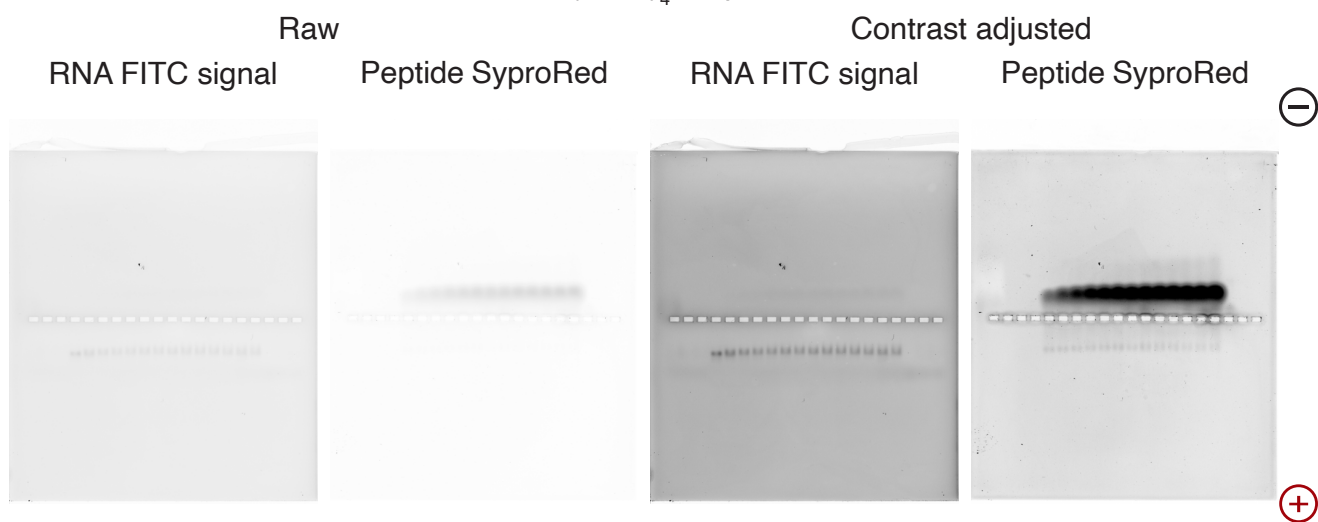

Supplement: Source Data Fig. 1 — Annotated complete gels Fig. 1 [file 41557_2022_890_MOESM2_ESM.pdf]

Fig 2a

$R_9$

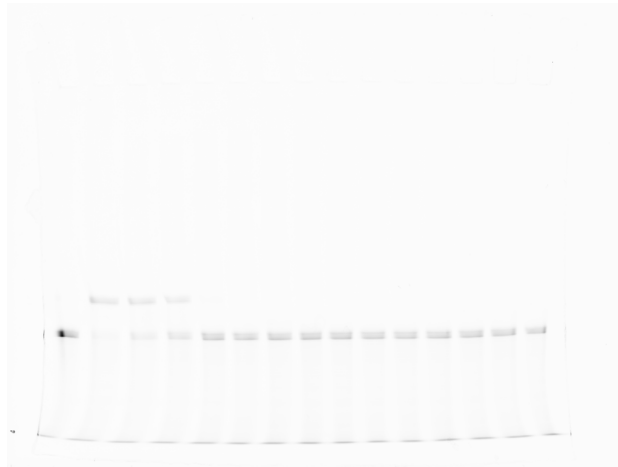

$K_9$

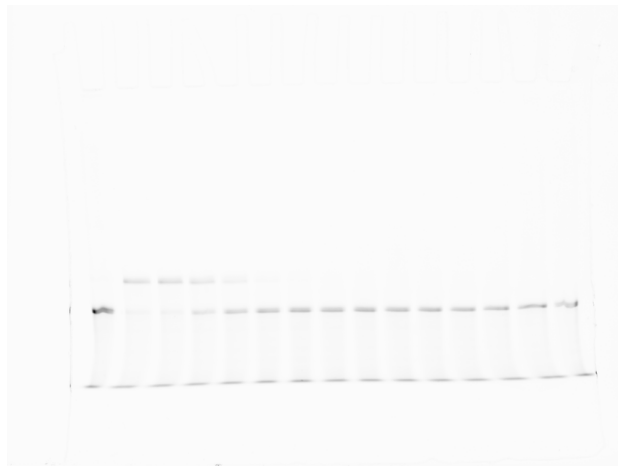

$(RGG)_4$

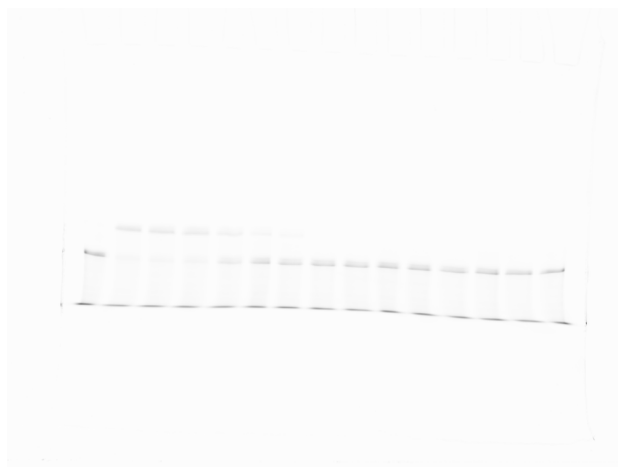

Supplement: Source Data Fig. 2 — Annotated complete gels Fig. 2f [file 41557_2022_890_MOESM4_ESM.pdf]
